# Risk factors for herpes simplex virus type-1 infection and reactivation: Cross-sectional studies among EPIC-Norfolk participants

**DOI:** 10.1371/journal.pone.0215553

**Published:** 2019-05-09

**Authors:** Harriet Forbes, Ben Warne, Lars Doelken, Nicole Brenner, Tim Waterboer, Robert Luben, Nicholas J. Wareham, Charlotte Warren-Gash, Effrossyni Gkrania-Klotsas

**Affiliations:** 1 Epidemiology and Population Health, London School of Hygiene and Tropical Medicine, London, United Kingdom; 2 Department of Medicine, University of Cambridge, Cambridge, United Kingdom; 3 Institute for Virology and Immunobiology, University of Wurzburg, Wurzburg, Germany; 4 NHS Blood and Transplant, Cambridge, United Kingdom; 5 Infections and Cancer Epidemiology, German Cancer Research Center (DKFZ), Heidelberg, Germany; 6 EPIC University Strangeways Research Laboratory, Cambridge, United Kingdom; 7 Department of Public Health and Primary Care, School of Clinical Medicine, University of Cambridge, Cambridge, United Kingdom; University of St Andrews, UNITED KINGDOM

## Abstract

**Background:**

The prevalence of, and risk factors for, herpes simplex virus type-1 (HSV-1) infection and reactivation in older individuals are poorly understood.

**Methods:**

This is a prospective population-based study among community-dwelling individuals aged 40–79 years, followed from 1993, formed as a random subsample of the UK-based EPIC-Norfolk cohort. HSV-1 seropositivity was derived from immunoglobulin G measurements and frequent oro-labial HSV reactivation was self-reported. We carried out two cross-sectional studies using logistic regression to investigate childhood social and environmental conditions as risk factors for HSV-1 seropositivity and comorbidities as risk factors for apparent HSV oro-labial reactivation.

**Results:**

Of 9,929 participants, 6310 (63.6%) were HSV-1 IgG positive, and 870 (of 4,934 seropositive participants with reactivation data) experienced frequent oro-labial reactivation. Being born outside the UK/Ireland, contemporaneous urban living and having ≥4 siblings were risk factors for HSV-1 seropositivity. Ever diagnosed with kidney disease, but no other comorbidities, was associated with an increased risk of frequent HSV reactivation (adjOR 1.87, 95%CI: 1.02–3.40).

**Discussion:**

Apparent HSV-1 seropositivity and clinical reactivation are common within an ageing UK population. HSV-1 seropositivity is socially patterned while risk factors for oro-labial HSV reactivation are less clear. Further large studies of risk factors are needed to inform HSV-1 control strategies.

## Introduction

HSV-1 is one of eight herpesviruses that routinely infect humans.[1] It is thought to be transmitted via close contact in childhood and it establishes latency in sensory ganglia. HSV-1 reactivation causes outbreaks of oro-labial, oropharyngeal, or increasingly, genital ulcers,[2] but can also be asymptomatic. Oro-labial ulcers can also be caused by HSV-2 reactivation[3] and the two viruses are clinically indistinguishable. However, oro-labial HSV-2 reactivation is very infrequent.[3] The prevalence of HSV-1 infection varies by age, time and geographic setting, with European seroprevalence estimates ranging from around 50–80%.[4] Following infection only around 30% of individuals with serologic evidence of HSV-1 experience clinical reactivation[5] but the factors involved in infection susceptibility and control of latent infection are poorly understood.

There is a growing interest in the role of chronic viruses in the pathogenesis of cardiovascular and neurodegenerative disorders affecting older individuals.[6, 7] Routinely collected health data are increasingly being used to assess the effects of such viruses on chronic diseases. However, HSV-1 is poorly recorded in routine health data so there is a need for population-based cohorts with biological and social measures to provide updated estimates of infection and reactivation. This will help both to plan public health interventions to control HSV-1 infection and guide the design of research studies of HSV-1 as a risk factor for other conditions.

Using data from a community-dwelling, ageing UK cohort, we therefore aimed to investigate the effect of childhood environmental and social conditions on the risk of HSV-1 seropositivity at a single time point in adulthood, alongside the effects of common clinical exposures and comorbidities of middle to later life on the risk of frequent self-reported HSV reactivation, among those infected with HSV-1.

## Methods

### Ethics statement

All volunteers gave written informed consent, and the study was approved by the Norfolk Research Ethics Committee (98CN01) and LSHTM ethics committee (14188).

### Data source

**Fig 1:**
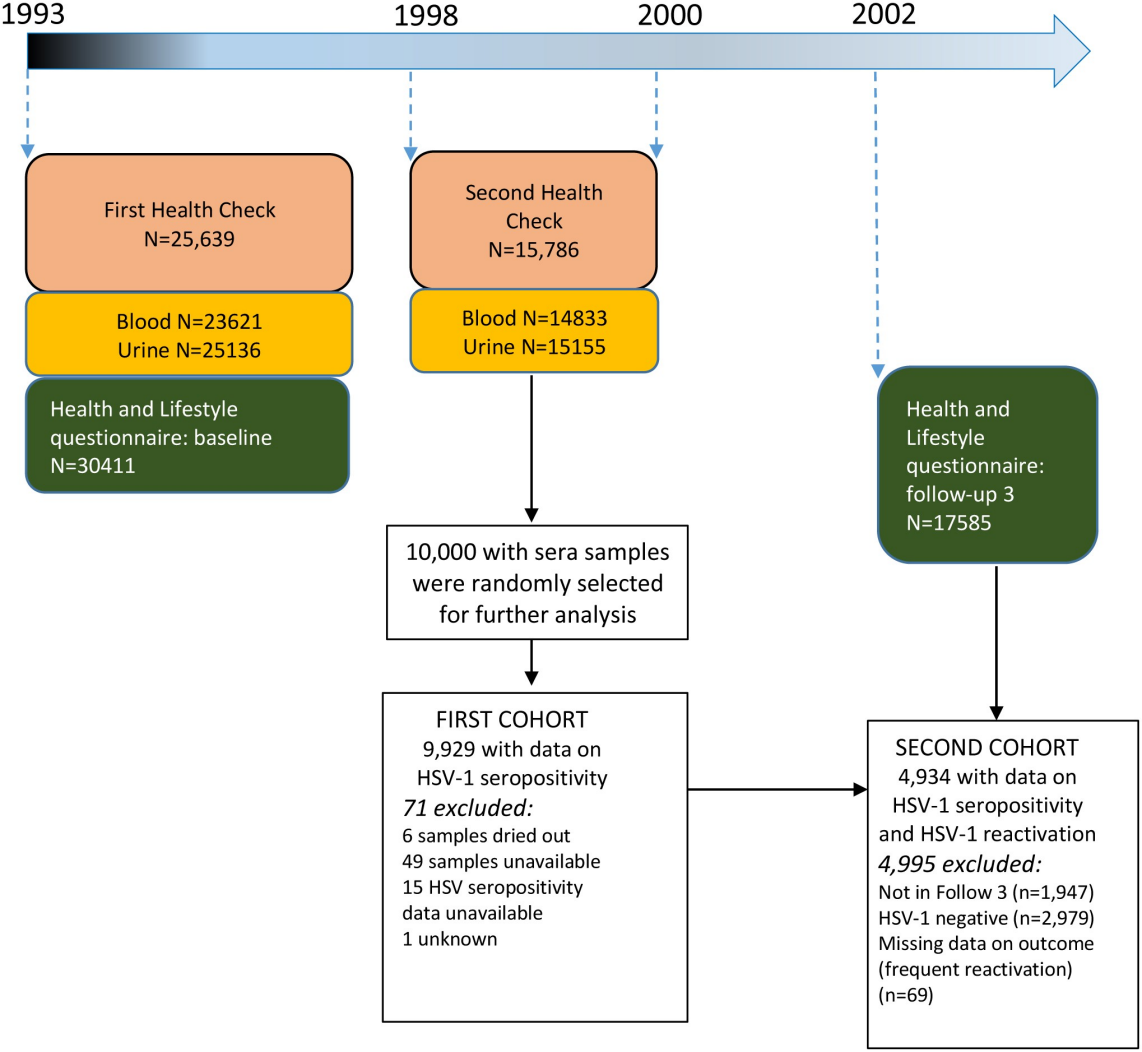
Timeline of EPIC Norfolk data and diagram of study participants utilized in this study.

The European Prospective Investigation of Cancer and Nutrition (EPIC) study design and methods have been described in detail elsewhere.[8–10] In brief, EPIC is a multi-center prospective cohort study and the EPIC-Norfolk cohort was recruited from the county of Norfolk in the East of England.[8] Individuals aged between 40 and 79 years registered at participating primary care practices were asked to join. Of 77,630 individuals invited to participate, 30,445 (39.2%) consented and 25,639 completed the first health check. Data collection is ongoing, with information gathered through self-reported questionnaires, health checks, biological samples and linked electronic databases. A time line of the EPIC Norfolk follow-up is shown in Fig 1.

All participants taking part in the first health check were invited to a second health check (from 1998 onwards) and 15,786/25,639 (61.6%) of the original cohort attended. Blood and urine samples were taken (described in detail previously[11]). From participants with available sera, 10,000 samples were randomly selected for further analysis, including characterizing HSV-1 and HSV-2 seropositivity (see Fig 1). Following the second health check, a third health and lifestyle questionnaire was sent between 2002 and 2004 (herein referred to as the Follow 3 questionnaire).

### Outcome definitions

The primary outcomes of interest were HSV-1 seropositivity and HSV-1 reactivation. Quantitative IgG antibody titres were determined using multiplex serology, an assay developed at the German Cancer Research Center (DKFZ) in Heidelberg. All analyses and quality control procedures were performed at DKFZ. HSV-1 seropositivity was defined by the presence of two positive antibody responses (above a pre-specified cut-off level) to the HSV-1 antigens gG and gD. The cut-off level was 100 mean fluorescence intensity (MFI) units for each antigen, based on visual inspection of turning points in percentile plots.[12] Both antigens are membrane glycoproteins important in virion attachment and entry into the host cell. The majority of the humoral response to HSV infection is targeted at these antigens. Seronegative meant the sample had either one or both negative responses to HSV-1 antigens. Among seropositive patients only, we created a categorical HSV-1 seropositivity variable by grouping participants into thirds of the antibody titre distribution (as there is no standard categorical definition of HSV-1 IgG titre levels). In the Follow 3 questionnaire participants were asked, “Are there any other infections which you commonly get more than once or twice a year?”, of which cold sores were specified as one option. This question therefore captures those with “frequent” HSV reactivation, defined as ≥2 reactivations per year.

In a further post-hoc sensitivity analysis we used a secondary definition of our outcomes, using single antigens to define HSV-1 (anti-gG response to HSV-1 is ≥300 MFI) and HSV-2 (anti-mgGunique response to HSV-2 ≥300 MFI) seropositivity; this was to reduce the theoretical risk of antibody cross-reactivity with the gD antigens. The 300 MFI cutoff was determined using Receiver Operating Characteristic curves produced by comparing the multiplex assay with the Enzygnost Anti-HSV/IgG (Dade Behring Marburg GmbH, Marburg, Germany) ELISA in 203 clinical samples processed at the Heidelberg diagnostic laboratory.

### Primary exposure definitions

All exposures below were self-reported, unless otherwise specified.

Childhood environmental and social conditions included: country of birth (as a proxy measure of background HSV-1 infection levels) at the second health check; socioeconomic status of household (with Townsend area deprivation indices derived from the 1991 census data, and categorised according to national Townsend quintiles from 1991[13]) and level of urbanisation at birth (using 1991 census data on level of urbanisation as a proxy); number of siblings (as a proxy measure of house hold density and degree of close contact) and measures of childhood health (including spending two weeks in hospital as a child and low birthweight) from the first health-check.

Common clinical comorbidities of middle to later life with an emphasis to any possible immunosuppressive medication use or condition were selected from the information collected via the Follow 3 questionnaire. Medications included corticosteroid use, other immunosuppressive medications (for example chemotherapy and methotrexate) and non-steroidal anti-inflammatory drugs, all derived from free-text data. Conditions included cancer (excluding non-melanoma skin cancer) externally derived from ICD-coded cancer registry data[8] prior to date of the third follow-up questionnaire. It also included the following conditions, which participants were asked whether their doctor had ever told them they had: rheumatoid arthritis, ulcerative colitis and/or Crohn’s disease, kidney disease (e.g. nephritis) and diabetes.

### Other exposures

We studied other exposures that could be associated with HSV reactivation based on previously published studies, including: age (in 10 year bands, as there was no evidence of non-linearity) and smoking status (current, former, never) derived from the second health check or Follow 3 questionnaire); body mass index (BMI: grouped according to World Health Organization categories), concentration of 25-hydroxyvitamin D3 (nmol/L) and HSV-2 seropositivity derived from the second health check (a positive gD antibody response to HSV-2 and positive to mgGunique antigen (both above a cut-off level of 100 MFI[12])); gender, ethnicity, highest educational qualification, having had an outdoor job (as a proxy measure of sunlight exposure), stress (from the question, “Has stress affected your health?”) derived from the first health check; and fatigue (from the question, “Do you feel tired all the time?”) from the Follow 3 questionnaire.

### Design and statistical analysis plan

We first described the characteristics of each cohort of interest. The first cohort comprised all those with HSV-1 serology data; the second cohort comprised HSV-1 seropositive individuals with data on oro-labial HSV reactivation. We explored whether the study participants in each cohort were representative of the main EPIC Norfolk cohort, in terms of age, gender, anthropometry, smoking status and blood pressure at baseline. We went on to carry out two cross sectional studies to understand the determinants of HSV-1 infection and frequent HSV reactivation.

The first study assessed the determinants of HSV-1 infection in the first cohort. The primary exposures of interest were childhood environmental and social conditions. Logistic regression was used to generate crude odds ratios to estimate the strength of association of each exposure with HSV-1 seropositivity, as well as adjusted odds ratios accounting for potential confounders. Our first model adjusted for ethnicity and gender, according to our causal assumptions (see causal diagram[14] in S1 Fig). We did not adjust for number of siblings because this may lie on the causal pathway. In a sensitivity analysis we additionally adjusted for HSV-2 seropositivity and age at the time of serology sampling.

The second cross sectional study examined the determinants of frequent HSV reactivation in the second cohort (see Fig 1). The primary exposures were common clinical comorbidities of middle to later life. Logistic regression was used to generate crude odds ratios to estimate the strength of association of each exposure with HSV reactivation as well as adjusted odds ratios, accounting for potential confounders. In our first model we adjusted for gender, age, ethnicity, SES, lifestyle risk factors (smoking, alcohol and BMI) and psychological stress, according to our causal assumptions (see causal diagram S1 Fig). In a sensitivity analysis we additionally adjusted for HSV-2 seropositivity, a proxy for UV light exposure and fatigue. *A-priori* we hypothesized age may modify the association between our primary exposures and frequent HSV reactivation, therefore we stratified our results by older and younger participants (using median age as the cut-off point). We also hypothesised frequency of cold sores and/or reporting of cold sores may differ by gender, therefore we stratified our results by gender.

All analyses were carried out in STATA, version 14.2 (College Station, TX).

## Results

In total 9,929 participants had HSV-1 serology data (first cohort) and 6,310 (64%) were seropositive for HSV-1 (Table 1). Of these seropositives, 4,934 participants (78%) had HSV reactivation data (second cohort—see Fig 1). The cohort was born between 1914 and 1957 and the median age of both cohorts was 62 years (IQR: 54–69) when serology specimens were collected. Compared to the main EPIC Norfolk cohort who attended the first health check, both the first and second cohort were more likely to be female and less likely to be >75 years, overweight and obese, current and former smokers and have high blood pressure (see S1 Table).

**Table 1:** Description of the first cohort, prevalence of HSV-1 infection and association between each variable and HSV-1 infection.

Variable (source of data)	Overall n (%)	Prevalence HSV-1+ n (%)	Unadjusted OR (95% CI)	Fully adjusted* OR (95% CI)	Sensitivity analysis: adjusted** OR (95% CI)
No of participants	9929 (100%)	6310 (63.6)	-	-	-
**Demographic characteristics**					
Gender (1HC)					
Males	4047 (40.8)	2531 (62.5)	1	1	1
Females	5882 (59.2)	3779 (64.2)	1.08 (0.99–1.17)	1.08 (0.99–1.17)	1.09 (1.00–1.19)
Age in years (2HC)					
40–49	659 (6.6)	361 (54.8)	1	1	1
50–59	3518 (35.4)	2084 (59.2)	1.20 (1.01–1.42)	1.21 (1.02–1.43)	1.24 (1.05–1.47)
60–69	3334 (33.6)	2181 (65.4)	1.56 (1.32–1.85)	1.58 (1.34–1.88)	1.65 (1.39–1.97)
70–79	2352 (23.7)	1640 (69.7)	1.90 (1.59–2.27)	1.94 (1.62–2.31)	2.02 (1.68–2.41)
80–89	66 (0.7)	44 (66.7)	1.65 (0.97–2.82)	1.68 (0.98–2.86)	1.75 (1.02–3.00)
Ethnicity (1HC)					
White	9863 (99.3)	6263 (63.5)	1	1	1
Other	66 (0.7)	47 (71.2)	1.42 (0.83–2.43)	1.43 (0.84–2.45)	1.52 (0.88–2.61)
Missing	33 (0.3)				
Highest educational qualification achieved (1HC)					
None	3316 (33.4)	2330 (70.3)	1	1	1
O-Level	1090 (11.0)	682 (62.6)	0.71 (0.61–0.82)	0.71 (0.61–0.82)	0.75 (0.65–0.87)
A-level	4110 (41.4)	2539 (61.8)	0.68 (0.62–0.75)	0.69 (0.62–0.76)	0.72 (0.65–0.80)
Degree or higher	1410 (14.2)	757 (53.7)	0.49 (0.43–0.56)	0.49 (0.43–0.56)	0.53 (0.46–0.60)
Missing	103 (1.0)				
**Health characteristics**					
BMI category (2HC)					
Underweight	41 (0.4)	21 (51.2)	0.66 (0.36–1.23)	0.64 (0.35–1.19)	0.68 (0.37–1.26)
Normal Weight	3623 (36.5)	2222 (61.3)	1	1	1
Overweight	4630 (46.6)	2953 (63.8)	1.11 (1.01–1.21)	1.13 (1.03–1.23)	1.09 (0.99–1.20)
Obese	1619 (16.3)	1104 (68.2)	1.35 (1.19–1.53)	1.36 (1.20–1.54)	1.29 (1.13–1.46)
Missing	16 (0.2)				
Smoking status (2HC)					
current smoker	739 (7.4)	514 (69.6)	1	1	1
former smoker	4201 (42.3)	2818 (67.1)	0.89 (0.75–1.06)	0.92 (0.77–1.09)	0.87 (0.73–1.04)
never smoked	4915 (49.5)	2927 (59.6)	0.64 (0.55–0.76)	0.64 (0.54–0.75)	0.64 (0.54–0.76)
Missing	74 (0.8)				
HSV-2 serostatus					
negative	9574 (96.4)	5964 (62.3)	1	1	1
positive	355 (3.6)	346 (97.5)	23.27 (11.99–45.16)	23.10 (11.90–44.83)	24.05 (12.38–46.70)
**Sociodemographic and childhood characteristics**					
Country of birth (2HC, F3)					
UK and Ireland	7702 (77.6)	4816 (62.5)	1	1	1
Other European country	96 (1.0)	73 (76.0)	1.90 (1.19–3.05)	1.88 (1.17–3.01)	1.70 (1.05–2.74)
US, Canada, Australia and NZ	40 (0.4)	19 (47.5)	0.54 (0.29–1.01)	0.54 (0.29–1.01)	0.46 (0.24–0.88)
Asia, S. America, Africa, Middle East	99 (1.0)	57 (57.6)	0.81 (0.54–1.21)	0.74 (0.48–1.13)	0.67 (0.43–1.04)
Missing	1992 (20.1)				
Level of urbanisation (1991 census data)					
Urban	4536 (45.7)	2968 (65.4)	1	1	1
Town	2159 (21.7)	1355 (62.8)	0.89 (0.80–0.99)	0.89 (0.80–0.99)	0.89 (0.80–0.99)
Village	2319 (23.4)	1465 (63.2)	0.91 (0.82–1.01)	0.91 (0.82–1.01)	0.93 (0.84–1.03)
Hamlet	910 (9.2)	519 (57.0)	0.70 (0.61–0.81)	0.70 (0.61–0.81)	0.73 (0.63–0.84)
Missing	5 (0.05)				
Townsend quintile (1HC)¹					
Q1 (most affluent)	5667 (57.1)	3555 (62.7)	1	1	1
Q2	2518 (25.4)	1591 (63.2)	1.02 (0.93–1.12)	1.02 (0.92–1.12)	1.02 (0.93–1.13)
Q3	1077 (10.8)	690 (64.1)	1.06 (0.92–1.21)	1.06 (0.92–1.21)	1.06 (0.93–1.22)
Q4	553 (5.6)	385 (69.6)	1.36 (1.13–1.64)	1.35 (1.12–1.64)	1.32 (1.09–1.60)
Q5 (most deprived)	84 (0.8)	70 (83.3)	2.97 (1.67–5.29)	2.97 (1.67–5.28)	2.97 (1.66–5.30)
Missing	30 (0.3)				
Number of siblings (1HC)					
0	1204 (12.1)	713 (59.2)	1.03 (0.90–1.19)	1.03 (0.90–1.19)	1.02 (0.88–1.18)
1	2398 (24.2)	1402 (58.5)	1	1	1
2	2141 (21.6)	1309 (61.1)	1.12 (0.99–1.26)	1.12 (0.99–1.26)	1.10 (0.97–1.24)
3	1372 (13.8)	867 (63.2)	1.22 (1.06–1.40)	1.22 (1.06–1.40)	1.21 (1.06–1.39)
4 or more	2814 (28.3)	2019 (71.7)	1.80 (1.61–2.03)	1.80 (1.60–2.02)	1.76 (1.56–1.97)
Spent 2+ weeks in a hospital during childhood (HLEQ baseline)					
No	6746 (67.9)	4237 (62.8)	1	1	1
Yes	1919 (19.3)	1245 (64.9)	1.09 (0.98–1.22)	1.10 (0.99–1.22)	1.07 (0.96–1.20)
Missing	1264 (12.7)				
Low birth weight (1HC)					
No	3897 (39.2)	2416 (62.0)	1	1	1
Yes (<2.5kg)	532 (5.4)	338 (63.5)	1.07 (0.88–1.29)	1.07 (0.88–1.29)	1.06 (0.87–1.29)
Missing	5500 (55.4)				

*Adjusted for ethnicity and sex.

**Adjusted for ethnicity, sex, age and HSV-2 serostatus.

¹Split according to cut-offs for England and Wales 1991. 1HC = First health check, 2HC = Second health check, F3 = Follow 3, HLEQ = Health and lifestyle questionnaire.

The risk of HSV-1 seropositivity (Table 1) was almost 2-fold greater for those born in another European country (compared to the UK and Ireland), 10–20% lower for those residing in towns or hamlets at the time of 1991 census (compared to urban areas), almost 3-fold greater for those in the lowest socioeconomic quintile (compared to the highest), and almost 2-fold greater for those with four or more siblings (compared to one). Self-reported childhood illness (warranting ≥2 weeks in hospital) was weakly associated with increased risk of HSV-1 seropositivity (adjOR 1.10, 95%CI 0.99–1.22). There was no evidence that having a low birthweight (<2.5kg) was associated with increased risk of HSV-1 seropositivity, though this variable had a considerable amount of missing data (55.4%). Being older, female, having lower educational achievement, being obese and a current or former smoker were associated with increased risk of HSV-1 seropositivity.

Of the 6,310 participants identified as HSV-1 seropositive, 4,934 (78%) had available information on oro-labial reactivation. Of these, 870 (17.6%) reportedly experienced frequent HSV reactivation (Table 2). Table 2 shows the association between frequent HSV reactivation and exposures of interest. Kidney disease (reported by 61 participants) was associated with an almost 2-fold increased risk of frequent reactivation (adjOR 1.87, 95%CI 1.02–3.40). No other medical conditions or medications were associated with frequent reactivation. There was some evidence that having held an outdoor job (reported by 729 participants) was associated with HSV reactivation (adjOR 1.33, 95% CI 1.06–1.67). However, concentration of Vitamin D3 was not associated. Higher levels of fatigue and stress were associated with an increased risk of frequent HSV reactivation. There was some weak evidence that HSV-2 seropositivity was associated with reduced risk of frequent HSV reactivation (adjOR 0.69, 95%CI 0.47–1.02).

**Table 2:** Description of the second cohort of HSV-1 positive individuals, prevalence of HSV reactivation and association between each variable and HSV reactivation.

		Overall n (%)	Prevalence HSV reactivation n (%)	Unadjusted OR (95% CI)	Fully adjusted* OR (95% CI)	Sensitivity analysis: adjusted** OR (95% CI)
Number of participants		4934 (100%)	870 (17.6)			
**Demographic characteristics**						
Age in years (F3)	40–49	112 (2.3)	19 (17.0)	1	1	1
	50–59	1164 (23.6)	238 (20.4)	1.26 (0.75–2.10)	1.19 (0.69–2.06)	1.24 (0.70–2.18)
	60–69	1700 (34.5)	298 (17.5)	1.04 (0.63–1.73)	1.02 (0.59–1.77)	1.07 (0.61–1.88)
	70–79	1550 (31.4)	261 (16.8)	0.99 (0.59–1.65)	1.03 (0.60–1.79)	1.09 (0.62–1.92)
	80–89	397 (8.0)	50 (12.6)	0.71 (0.40–1.25)	0.66 (0.35–1.23)	0.68 (0.36–1.28)
Gender (1HC)	Males	1940 (39.3)	321 (16.5)	1	1	1
	Females	2994 (60.7)	549 (18.3)	1.13 (0.97–1.32)	1.07 (0.90–1.27)	1.11 (0.93–1.32)
Ethnicity (1HC)	White	4899 (99.3)	866 (17.7)	1	1	1
	Other	35 (0.7)	4 (11.4)	0.60 (0.21–1.71)	0.72 (0.21–2.46)	0.74 (0.21–2.59)
Education level (1HC)	None	1722 (34.9)	296 (17.2)	1	1	1
	O-Level	547 (11.1)	110 (20.1)	1.21 (0.95–1.55)	1.08 (0.83–1.41)	1.07 (0.82–1.41)
	A-level	2027 (41.1)	355 (17.5)	1.02 (0.86–1.21)	0.97 (0.80–1.17)	0.98 (0.81–1.18)
	Degree or higher	636 (12.9)	109 (17.1)	1.00 (0.78–1.27)	0.90 (0.69–1.18)	0.94 (0.72–1.23)
	Missing	2 (0.04)				
Townsend quintile (1HC)¹	Q1 (most affluent)	2824 (57.2)	504 (17.8)	1	1	1
	Q2	1246 (25.3)	220 (17.7)	0.99 (0.83–1.18)	0.97 (0.80–1.17)	0.95 (0.79–1.15)
	Q3	530 (10.7)	80 (15.1)	0.82 (0.63–1.06)	0.81 (0.61–1.06)	0.78 (0.59–1.03)
	Q4	278 (5.6)	58 (20.9)	1.21 (0.89–1.65)	1.28 (0.92–1.78)	1.28 (0.92–1.79)
	Q5 (most deprived)	43 (0.9)	7 (16.3)	0.90 (0.40–2.02)	1.25 (0.54–2.91)	1.33 (0.57–3.12)
	Missing	13 (0.3)				
**Immunosuppressive medications and conditions**						
Corticosteroids (F3)	No	4307 (87.3)	771 (17.9)	1	1	1
	Yes	193 (3.9)	34 (17.6)	0.98 (0.67–1.43)	0.94 (0.62–1.42)	0.88 (0.58–1.34)
	Missing	434 (8.8)				
Immunosuppressive medications (F3)	No	4875 (98.8)	860 (17.6)	1	1	1
	Yes	59 (1.2)	10 (16.9)	0.95 (0.48–1.89)	0.89 (0.41–1.91)	0.83 (0.39–1.80)
NSAIDs (F3)	No	3683 (74.6)	663 (18.0)	1	1	1
	Yes	1251 (25.4)	207 (16.5)	0.90 (0.76–1.07)	0.94 (0.78–1.14)	0.92 (0.76–1.11)
Arthritis (2HC)	No	3026 (61.3)	523 (17.3)	1	1	1
	Yes	1509 (30.6)	273 (18.1)	1.06 (0.90–1.24)	1.16 (0.96–1.39)	1.09 (0.90–1.32)
	Missing	399 (8.1)				
Ulcerative Colitis / Crohn`s Disease (F3)	No	4540 (92.0)	810 (17.8)	1	1	1
	Yes	75 (1.5)	14 (18.7)	1.06 (0.59–1.90)	1.00 (0.53–1.87)	1.00 (0.53–1.85)
	Missing	319 (6.5)				
Kidney Disease (F3)	No	4579 (92.8)	812 (17.7)	1	1	1
	Yes	61 (1.2)	17 (27.9)	1.79 (1.02–3.15)	1.87 (1.02–3.40)	1.87 (1.02–3.44)
	Missing	294 (6.0)				
Diabetes (2HC)	No	4749 (96.3)	837 (17.6)	1	1	1
	Yes	185 (3.7)	33 (17.8)	1.01 (0.69–1.49)	0.94 (0.60–1.46)	0.96 (0.62–1.51)
Cancer (from cancer registry data)	No	4416 (89.5)	788 (17.8)	1	1	1
	Yes	518 (10.5)	82 (15.8)	0.87 (0.68–1.11)	0.85 (0.65–1.12)	0.85 (0.65–1.12)
**Other known risk factors for HSV reactivation**						
UV light exposure: had an outdoor job (1HC)	No	4202 (85.2)	720 (17.1)	1	1	1
	Yes	729 (14.8)	149 (20.4)	1.24 (1.02–1.51)	1.33 (1.06–1.67)	1.33 (1.06–1.67)
	Missing	3 (0.1)				
Concentration of 25-Hydroxyvitamin D3 (nmol/L)(2HC)	Deficient (0–29)	507 (10.3)	73 (14.4)	0.76 (0.58–1.01)	0.79 (0.58–1.07)	0.79 (0.58–1.07)
	Insufficiency (30–49)	1492 (30.2)	254 (17.0)	0.93 (0.77–1.12)	1.02 (0.83–1.25)	1.02 (0.83–1.25)
	Adequate (50–69)	1602 (32.5)	289 (18.0)	1	1	1
	High (70–89)	887 (18.0)	177 (20.0)	1.13 (0.92–1.39)	1.17 (0.93–1.47)	1.17 (0.93–1.47)
	Undesirably high (90+)	428 (8.7)	73 (17.1)	0.93 (0.70–1.24)	0.98 (0.72–1.33)	0.98 (0.72–1.33)
	Missing	18 (0.4)				
Do you feel tired? (F3)	All of the time	112 (2.3)	27 (24.1)	1.50 (0.96–2.34)	1.31 (0.78–2.21)	1.30 (0.77–2.19)
	Most of the time	258 (5.2)	71 (27.5)	1.79 (1.33–2.41)	1.65 (1.19–2.28)	1.67 (1.20–2.31)
	A good bit of the time	551 (11.2)	107 (19.4)	1.14 (0.89–1.44)	1.02 (0.78–1.32)	1.01 (0.77–1.31)
	Some of the time	2052 (41.6)	359 (17.5)	1	1	1
	A little of the time	1617 (32.8)	272 (16.8)	0.95 (0.80–1.13)	0.95 (0.79–1.14)	0.95 (0.79–1.14)
	None of the time	294 (6.0)	30 (10.2)	0.54 (0.36–0.80)	0.55 (0.36–0.84)	0.55 (0.36–0.85)
	Missing	50 (1.0)				
Has stress affected your health? (1HC)	Not at all	1356 (27.5)	205 (15.1)	1	1	1
	A little	1869 (37.9)	338 (18.1)	1.24 (1.03–1.50)	1.22 (1.01–1.48)	1.17 (0.96–1.42)
	A moderate amount	747 (15.1)	150 (20.1)	1.41 (1.12–1.78)	1.39 (1.10–1.76)	1.28 (1.00–1.63)
	A great deal	363 (7.4)	76 (20.9)	1.49 (1.11–1.99)	1.45 (1.08–1.95)	1.28 (0.94–1.74)
	Missing	599 (12.1)				
**Health characteristics**						
BMI category (2HC)	Underweight	13 (0.3)	3 (23.1)	1.32 (0.36–4.83)	1.10 (0.23–5.29)	1.29 (0.27–6.24)
	Normal Weight	1772 (35.9)	328 (18.5)	1	1	1
	Overweight	2317 (47.0)	388 (16.7)	0.89 (0.75–1.04)	0.88 (0.73–1.05)	0.87 (0.73–1.04)
	Obese	826 (16.7)	151 (18.3)	0.98 (0.80–1.22)	0.99 (0.79–1.25)	0.96 (0.76–1.21)
	Missing	6 (0.1)				
Smoking status (F3)	Current smoker	371 (7.5)	50 (13.5)	0.69 (0.50–0.94)	0.61 (0.42–0.86)	0.56 (0.39–0.81)
	Former smoker	2293 (46.5)	399 (17.4)	0.93 (0.80–1.08)	0.94 (0.79–1.11)	0.93 (0.78–1.10)
	Never smoked	2244 (45.5)	415 (18.5)	1	1	1
	Missing	26 (0.5)				
HSV-2 infection	No	4671 (94.7)	835 (17.9)	1	1	1
	Yes	263 (5.3)	35 (13.3)	0.71 (0.49–1.01)	0.69 (0.47–1.02)	0.69 (0.47–1.03)
Level of HSV-1 IgG, in tertiles	Low	1644 (33.3)	192 (11.7)	1	1	1
	Medium	1645 (33.3)	342 (20.8)	1.98 (1.64–2.40)	2.10 (1.70–2.59)	2.08 (1.68–2.57)
	High	1645 (33.3)	336 (20.4)	1.94 (1.60–2.35)	2.11 (1.71–2.60)	2.09 (1.69–2.59)
	Missing	33 (0.3)				

*Adjusted for gender, age, ethnicity, SES, lifestyle factors (smoking and BMI) and psychological stress

**Additionally adjusted for UV light exposure, fatigue and HSV-2 status. NSAIDs: Non-steroidal anti-inflammatories. 1HC = First health check, 2HC = Second health check, F3 = Follow 3, HLEQ = Health and lifestyle questionnaire.

Stratified results by age and sex are given in the supplementary material (S2 and S3 Tables). Briefly, the increased risk of frequent HSV reactivation among participants with kidney disease was restricted to participants ≥65 years of age (adjOR2.66, 95%CI 1.14–6.21) and also females (adjOR2.43, 95%CI 1.23–4.81). By contrast, the increased risk of frequent HSV reactivation among those with an outdoor job was restricted to males. HSV-2 seropositivity was associated with a reduced risk of frequent HSV reactivation among females (adjOR 0.53, 95%CI:0.32–0.89), but not among males (adjOR 1.15, 95%CI:0.61–2.14).

In our post-hoc sensitivity analysis HSV-1 and HSV-2 were redefined using single antigens. Although the seroprevalence of HSV-1 increased markedly, the results for risk factors for HSV-1 seropositivity were broadly the same as the main analyses, aside from there being no evidence of a reduced risk of HSV-1 seropositivity for those living in a hamlet and the association between HSV-1 and HSV-2 prevalence reduced dramatically (Table 3). In the risk factors for HSV reactivation, the results were broadly similar, aside from there being a clearer dose-response relationship between increased level of HSV-1 IgG and increased risk of HSV reactivation (Table 4).

**Table 3:** Description of the first cohort, prevalence of HSV-1 infection defined using a single antigen and association between each variable and HSV-1 infection.

Variable (source of data)	Overall n (%)	Prevalence HSV-1+ n (%)	Unadjusted OR (95% CI)	Fully adjusted* OR (95% CI)	Sensitivity analysis: adjusted** OR (95% CI)
No of participants	9929 (100S)	9073 (91.4)	-	-	-
**Demographic characteristics**					
Gender (1HC)					
Males	4047 (40.8)	3676 (90.8)			
Females	5882 (59.2)	5397 (91.8)	1.12 (0.97–1.29)	1.12 (0.98–1.29)	1.14 (0.99–1.31)
Age in years (2HC)					
40–49	659 (6.6)	593 (90.0)	1.00	1.00	1.00
50–59	3518 (35.4)	3187 (90.6)	1.07 (0.81–1.42)	1.08 (0.81–1.42)	1.08 (0.82–1.43)
60–69	3334 (33.6)	3038 (91.1)	1.14 (0.86–1.51)	1.16 (0.87–1.54)	1.17 (0.88–1.55)
70–79	2352 (23.7)	2194 (93.3)	1.55 (1.14–2.09)	1.57 (1.16–2.13)	1.58 (1.17–2.14)
80–89	66 (0.7)	61 (92.4)	1.36 (0.53–3.50)	1.38 (0.53–3.55)	1.38 (0.54–3.56)
Ethnicity (1HC)					
White	9863 (99.3)	9012 (91.4)	1.00	1.00	1.00
Other	66 (0.7)	61 (92.4)	1.15 (0.46–2.87)	1.17 (0.47–2.91)	1.20 (0.48–3.00)
Missing	33 (0.3)				
Highest educational qualification achieved (1HC)					
None	3316 (33.4)	3087 (93.1)	1.00	1.00	1.00
O-Level	1090 (11.0)	1006 (92.3)	0.89 (0.69–1.15)	0.89 (0.68–1.15)	0.94 (0.72–1.22)
A-level	4110 (41.4)	3732 (90.8)	0.73 (0.62–0.87)	0.74 (0.62–0.88)	0.77 (0.64–0.91)
Degree or higher	1410 (14.2)	1246 (88.4)	0.56 (0.46–0.70)	0.57 (0.46–0.70)	0.60 (0.48–0.75)
Missing	103 (1.0)				
**Health characteristics**					
BMI category (2HC)					
Underweight	41 (0.4)	35 (85.4)	0.68 (0.28–1.62)	0.65 (0.27–1.56)	0.67 (0.28–1.61)
Normal Weight	3623 (36.5)	3246 (89.6)	1.00	1.00	1.00
Overweight	4630 (46.6)	4270 (92.2)	1.38 (1.18–1.60)	1.41 (1.21–1.65)	1.40 (1.20–1.63)
Obese	1619 (16.3)	1507 (93.1)	1.56 (1.25–1.95)	1.57 (1.26–1.96)	1.54 (1.23–1.92)
Missing	16 (0.2)				
Smoking status (2HC)					
current smoker	739 (7.4)	693 (93.8)	1.00	1.00	1.00
former smoker	4201 (42.3)	3920 (93.3)	0.93 (0.67–1.28)	0.97 (0.70–1.33)	0.93 (0.67–1.29)
never smoked	4915 (49.5)	4393 (89.4)	0.56 (0.41–0.76)	0.55 (0.40–0.75)	0.55 (0.40–0.75)
Missing	74 (0.8)				
HSV-2 serostatus†					
negative	9649 (97.2)	8804 (91.2)	1.00	1.00	1.00
positive	280 (2.8)	269 (96.1)	2.35 (1.28–4.31)	2.31 (1.26–4.24)	2.32 (1.26–4.26)
**Health characteristics**					
Country of birth (2HC, F3)					
UK and Ireland	7702 (77.6)	6998 (90.9)	1.00	1.00	1.00
Other European country	96 (1.0)	92 (95.8)	2.31 (0.85–6.32)	2.28 (0.84–6.22)	2.12 (0.78–5.81)
US, Canada, Australia and NZ	40 (0.4)	38 (95.0)	1.91 (0.46–7.94)	1.92 (0.46–7.99)	1.86 (0.45–7.75)
Asia, S. America, Africa, Middle East	99 (1.0)	91 (91.9)	1.14 (0.55–2.37)	1.11 (0.52–2.39)	1.07 (0.50–2.30)
Missing	1992 (20.1)				
Level of urbanisation (1991 census data)					
Urban	4536 (45.7)	4154 (91.6)	1.00	1.00	1.00
Town	2159 (21.7)	1961 (90.8)	0.91 (0.76–1.09)	0.91 (0.76–1.09)	0.91 (0.76–1.09)
Village	2319 (23.4)	2121 (91.5)	0.99 (0.82–1.18)	0.99 (0.83–1.18)	1.00 (0.84–1.20)
Hamlet	910 (9.2)	832 (91.4)	0.98 (0.76–1.27)	0.98 (0.76–1.27)	1.01 (0.79–1.31)
Missing	5 (0.05)				
Townsend quintile (1HC)¹					
Q1 (most affluent)	5667 (57.1)	5162 (91.1)	1.00	1.00	1.00
Q2	2518 (25.4)	2293 (91.1)	1.00 (0.85–1.18)	1.00 (0.85–1.17)	1.00 (0.84–1.17)
Q3	1077 (10.8)	992 (92.1)	1.14 (0.90–1.45)	1.14 (0.90–1.45)	1.14 (0.90–1.45)
Q4	553 (5.6)	516 (93.3)	1.36 (0.97–1.93)	1.35 (0.96–1.91)	1.32 (0.93–1.87)
Q5 (most deprived)	84 (0.8)	82 (97.6)	4.01 (0.98–16.36)	4.02 (0.98–16.38)	3.99 (0.98–16.28)
Missing	30 (0.3)				
Number of siblings (1HC)					
0	1204 (12.1)	1060 (88.0)	0.90 (0.72–1.11)	0.90 (0.72–1.11)	0.89 (0.72–1.11)
1	2398 (24.2)	2138 (89.2)	1.00	1.00	1.00
2	2141 (21.6)	1954 (91.3)	1.27 (1.04–1.55)	1.27 (1.04–1.55)	1.25 (1.03–1.53)
3	1372 (13.8)	1260 (91.8)	1.37 (1.08–1.73)	1.37 (1.08–1.72)	1.35 (1.07–1.70)
4 or more	2814 (28.3)	2661 (94.6)	2.12 (1.72–2.60)	2.12 (1.72–2.60)	2.06 (1.67–2.54)
Spent 2+ weeks in a hospital during childhood (HLEQ baseline)					
No	6746 (67.9)	6143 (91.1)	1.00	1.00	1.00
Yes	1919 (19.3)	1766 (92.0)	1.13 (0.94–1.36)	1.14 (0.95–1.37)	1.13 (0.94–1.36)
Missing	1264 (12.7)				
Low birth weight (1HC)					
No	3897 (39.2)	3576 (91.8)	1.00	1.00	1.00
Yes (<2.5kg)	532 (5.4)	493 (92.7)	1.13 (0.80–1.60)	1.16 (0.82–1.64)	1.15 (0.81–1.63)
Missing	5500 (55.4)				

*Adjusted for ethnicity and sex.

**Adjusted for ethnicity, sex, age and HSV-2 serostatus.

¹Split according to cut-offs for England and Wales 1991.

1HC = First health check, 2HC = Second health check, F3 = Follow 3, HLEQ = Health and lifestyle questionnaire.

†Defined through a single antigen

**Table 4:** Description of the second cohort of HSV-1 positive patients (defined by a single antigen), prevalence of HSV reactivation and association between each variable and HSV reactivation.

		Overall n (%)	Prevalence HSV reactivation n (%)	Unadjusted OR (95% CI)	Fully adjusted* OR (95% CI)	Sensitivity analysis: adjusted** OR (95% CI)
Number of participants		7170 (100%)	887 (12.1)			
Age in years (F3)	40–49	179 (2.5)	22 (12.3)	1.00	1.00	1.00
	50–59	1868 (26.1)	241 (12.9)	1.06 (0.66–1.68)	1.05 (0.64–1.72)	1.12 (0.68–1.86)
	60–69	2456 (34.3)	306 (12.5)	1.02 (0.64–1.61)	1.03 (0.63–1.68)	1.11 (0.67–1.83)
	70–79	2120 (29.6)	263 (12.4)	1.01 (0.64–1.61)	1.09 (0.67–1.79)	1.18 (0.71–1.96)
	80–89	527 (7.4)	51 (9.7)	0.76 (0.45–1.30)	0.74 (0.42–1.31)	0.79 (0.44–1.41)
Gender (1HC)	Males	2854 (39.8)	330 (11.6)			
	Females	4316 (60.2)	557 (12.9)	1.13 (0.98–1.31)	1.10 (0.94–1.30)	1.14 (0.96–1.35)
Ethnicity (1HC)	White	7123 (99.3)	883 (12.4)	1.00	1.00	1.00
	Other	47 (0.7)	4 (8.5)	0.66 (0.24–1.84)	0.68 (0.21–2.26)	0.73 (0.22–2.43)
Education level (1HC)	None	2305 (32.1)	299 (13.0)	1.00	1.00	1.00
	O-Level	798 (11.1)	114 (14.3)	1.12 (0.89–1.41)	1.00 (0.78–1.30)	1.00 (0.77–1.29)
	A-level	3003 (41.9)	363 (12.1)	0.92 (0.78–1.09)	0.89 (0.74–1.06)	0.90 (0.75–1.07)
	Degree or higher	1062 (14.8)	111 (10.5)	0.78 (0.62–0.99)	0.73 (0.57–0.94)	0.75 (0.59–0.97)
	Missing	2 (0.04)				
Townsend quintile (1HC)¹	Q1 (most affluent)	4143 (57.8)	516 (12.5)	1.00	1.00	1.00
	Q2	1809 (25.2)	224 (12.4)	0.99 (0.84–1.17)	0.96 (0.81–1.16)	0.95 (0.79–1.14)
	Q3	764 (10.7)	79 (10.3)	0.81 (0.63–1.04)	0.79 (0.60–1.03)	0.77 (0.59–1.01)
	Q4	383 (5.3)	59 (15.4)	1.28 (0.96–1.71)	1.30 (0.95–1.78)	1.29 (0.94–1.77)
	Q5 (most deprived)	50 (0.7)	7 (14.0)	1.14 (0.51–2.56)	1.48 (0.65–3.37)	1.60 (0.70–3.67)
	Missing	13 (0.3)				
Corticosteroids (F3)	No	6287 (87.7)	786 (12.5)	1.00	1.00	1.00
	Yes	283 (3.9)	36 (12.7)	1.02 (0.71–1.46)	0.95 (0.64–1.40)	0.90 (0.61–1.33)
	Missing	434 (8.8)				
Immunosuppressive medications (F3)	No	7079 (98.7)	876 (12.4)	1.00	1.00	1.00
	Yes	91 (1.3)	11 (12.1)	0.97 (0.52–1.84)	0.89 (0.44–1.80)	0.82 (0.40–1.66)
NSAIDs (F3)	No	5391 (75.2)	677 (12.6)	1.00	1.00	1.00
	Yes	1779 (24.8)	210 (11.8)	0.93 (0.79–1.10)	0.96 (0.80–1.15)	0.93 (0.78–1.12)
Arthritis (2HC)	No	4492 (62.6)	533 (11.9)	1.00	1.00	1.00
	Yes	2125 (29.6)	279 (13.1)	1.12 (0.96–1.31)	1.16 (0.98–1.39)	1.11 (0.93–1.33)
	Missing	399 (8.1)				
Ulcerative Colitis / Crohn`s Disease (F3)	No	6634 (92.5)	826 (12.5)	1.00	1.00	1.00
	Yes	104 (1.5)	15 (14.4)	1.19 (0.68–2.06)	1.16 (0.65–2.08)	1.18 (0.66–2.10)
	Missing	319 (6.5)				
Kidney Disease (F3)	No	6680 (93.2)	828 (12.4)	1.00	1.00	1.00
	Yes	87 (1.2)	17 (19.5)	1.72 (1.01–2.93)	1.81 (1.03–3.18)	1.76 (1.00–3.11)
	Missing	294 (6.0)				
Diabetes (2HC)	No	6915 (96.4)	854 (12.3)	1.00	1.00	1.00
	Yes	255 (3.6)	33 (12.9)	1.05 (0.73–1.53)	0.99 (0.65–1.50)	0.99 (0.65–1.51)
Cancer (from cancer registry data)	No	6425 (89.6)	802 (12.5)	1.00	1.00	1.00
	Yes	745 (10.4)	85 (11.4)	0.90 (0.71–1.15)	0.87 (0.67–1.13)	0.86 (0.66–1.13)
UV light exposure: had an outdoor job (1HC)	No	6104 (85.1)	734 (12.0)	1.00	1.00	1.00
	Yes	1063 (14.8)	152 (14.3)	1.22 (1.01–1.47)	1.30 (1.05–1.62)	1.30 (1.05–1.62)
	Missing	3 (0.1)				
Concentration of 25-Hydroxyvitamin D3 (nmol/L)(2HC)	Deficient (0–29)	769 (10.7)	77 (10.0)	0.78 (0.60–1.01)	0.77 (0.58–1.03)	0.77 (0.58–1.03)
	Insufficiency (30–49)	2171 (30.3)	257 (11.8)	0.94 (0.78–1.12)	1.01 (0.83–1.22)	1.01 (0.83–1.22)
	Adequate (50–69)	2337 (32.6)	293 (12.5)	1.00	1.00	1.00
	High (70–89)	1273 (17.8)	183 (14.4)	1.17 (0.96–1.43)	1.21 (0.97–1.50)	1.21 (0.97–1.50)
	Undesirably high (90+)	596 (8.3)	73 (12.2)	0.97 (0.74–1.28)	1.01 (0.75–1.36)	1.01 (0.75–1.36)
	Missing	18 (0.4)				
Do you feel tired? (F3)	All of the time	167 (2.3)	27 (16.2)	1.39 (0.91–2.13)	1.19 (0.73–1.96)	1.18 (0.72–1.94)
	Most of the time	388 (5.4)	74 (19.1)	1.70 (1.29–2.24)	1.52 (1.12–2.06)	1.53 (1.13–2.07)
	A good bit of the time	778 (10.9)	111 (14.3)	1.20 (0.95–1.51)	1.09 (0.85–1.40)	1.09 (0.85–1.39)
	Some of the time	2986 (41.6)	364 (12.2)	1.00	1.00	1.00
	A little of the time	2362 (32.9)	277 (11.7)	0.96 (0.81–1.13)	0.97 (0.81–1.16)	0.97 (0.81–1.16)
	None of the time	411 (5.7)	30 (7.3)	0.57 (0.38–0.84)	0.58 (0.38–0.89)	0.59 (0.39–0.90)
	Missing	50 (1.0)				
Has stress affected your health? (1HC)	Not at all	1970 (27.5)	206 (10.5)	1.00	1.00	1.00
	A little	2693 (37.6)	347 (12.9)	1.27 (1.05–1.52)	1.26 (1.04–1.51)	1.21 (1.01–1.47)
	A moderate amount	1140 (15.9)	156 (13.7)	1.36 (1.09–1.70)	1.34 (1.07–1.68)	1.23 (0.98–1.55)
	A great deal	520 (7.3)	79 (15.2)	1.53 (1.16–2.03)	1.50 (1.13–1.99)	1.34 (1.00–1.80)
	Missing	599 (12.1)				
BMI category (2HC)	Underweight	25 (0.3)	3 (12.0)	0.94 (0.28–3.15)	0.75 (0.17–3.29)	0.79 (0.18–3.48)
	Normal Weight	2610 (36.4)	331 (12.7)	1.00	1.00	1.00
	Overweight	3383 (47.2)	400 (11.8)	0.92 (0.79–1.08)	0.91 (0.77–1.08)	0.90 (0.75–1.06)
	Obese	1143 (15.9)	153 (13.4)	1.06 (0.87–1.31)	1.03 (0.83–1.29)	0.99 (0.79–1.24)
	Missing	6 (0.1)				
Smoking status (F3)	Current smoker	502 (7.0)	51 (10.2)	0.79 (0.58–1.07)	0.71 (0.51–1.00)	0.67 (0.47–0.95)
	Former smoker	3241 (45.2)	404 (12.5)	0.99 (0.86–1.15)	1.00 (0.85–1.18)	0.99 (0.84–1.16)
	Never smoked	3391 (47.3)	425 (12.5)	1.00	1.00	1.00
	Missing	26 (0.5)				
HSV-2 infection†	No	6964 (97.1)	864 (12.4)	1.00	1.00	1.00
	Yes	206 (2.9)	23 (11.2)	0.89 (0.57–1.38)	0.87 (0.54–1.39)	0.87 (0.54–1.40)
Level of HSV-1 IgG, in tertiles	Low	2390 (33.3)	40 (1.7)	1.00	1.00	1.00
	Medium	2390 (33.3)	341 (14.3)	9.78 (7.01–13.64)	10.85 (7.52–15.66)	10.84 (7.51–15.64)
	High	2390 (33.3)	506 (21.2)	15.78 (11.37–21.89)	18.08 (12.59–25.97)	18.28 (12.72–26.27)
	Missing	33 (0.3)				

*Adjusted for gender, age, ethnicity, SES, lifestyle factors (smoking and BMI) and psychological stress

**Additionally adjusted for UV light exposure, fatigue and HSV-2 status. NSAIDs: Non-steroidal anti-inflammatories. 1HC = First health check, 2HC = Second health check, F3 = Follow 3, HLEQ = Health and lifestyle questionnaire.

†Defined through a single antigen.

¹Split according to cut-offs for England and Wales 1991.

## Discussion

In this cohort of older community-dwelling individuals from England, we found a high prevalence of HSV-1 seropositivity (63.6%) and frequent apparent reactivation (17.6%). We found that childhood environmental and social conditions were associated with greater risk of HSV-1 seropositivity, specifically poorer socioeconomic status, having more siblings and living in an urban area. Common comorbidities were not associated with HSV reactivation with the exception of kidney disease, which was associated with a 2-fold increased risk, an association which was restricted to older females (≥65 years). Fatigue and stress increased the risk of HSV reactivation, as did having an outdoor job (an association restricted to males).

The prevalence of HSV-1 seropositivity in this cohort was similar to estimates in England and Wales during the 1990s[4] as well as the proportion found to be seropositive in recently released data from UK Biobank (~70%), which has a comparable population.[15] Data on HSV-1 reactivation are less clear, with an estimated 12%[5] of HSV-1 infected people experiencing two or more reactivations per year. The figure may be slightly higher in our study, due to an older cohort and differing definitions of reactivation (one rather than two or more cold sores per year).[16]

Many of the risk factors associated with HSV-1 seropositivity identified in this study are in keeping with previous research, including increasing age [1, 17–19], lower educational level and socioeconomic status[20]. Our study found increased prevalence with age up to 70 years, after which the risk declined: this decline may reflect a cohort effect (that participants from older generations may have been exposed to HSV-1 less than younger generations or that those who reached 80 years are generally healthier and less susceptible to HSV-1). Non-white ethnicity[1, 20, 21] has been associated with greater risk of HSV-1 infection, however this cohort was predominately white (99.3%) and therefore unable to examine the effect of ethnic group. Literature regarding gender is conflicting, with some papers suggesting female gender[20, 22, 23] is associated with greater risk of HSV-1 infection, whilst others report no association.[1, 24] The small increased risk of HSV-1 seropositivity with female sex we observed may reflect different behaviours increasing opportunity for exposure, such as greater childcare responsibilities. It may also reflect different immune response among females, or greater genetic susceptibility to infection.

This is one of few studies to demonstrate that urban living and increased number of siblings may increase the risk of HSV-1 seropositivity,[20] perhaps by increasing the opportunity for close-contact. These factors may also reflect an overall lower socioeconomic status; having a lower educational level, being overweight and having smoked were all associated with HSV-1 seropositivity, perhaps again reflecting lower socioeconomic status during childhood.

HSV-2 seropositivity was very strongly associated with HSV-1 seropositivity. This may be as a result of similar behaviours putting individuals at risk of these viruses, or perhaps a genetic predisposition to herpes simplex viruses. The association reduced significantly when defining HSV-2 seropositivity by mgGunique alone, suggesting in our primary analyses some of the association was due to antibody cross-reactivity. However there was still strong evidence of an association, suggesting shared route of exposure or shared genetic predisposition.

Hypothesized factors for HSV-1 reactivation include immunosuppression[21] and there is evidence that reactivation of other herpesviruses is more common in immunosuppressed patients, such as those with chronic kidney disease.[25, 26] Our finding that older women with kidney disease are at greater risk of experiencing frequent reactivation supports this hypothesis. However, kidney disease was a self-reported variable, derived from a question, “Has your doctor had ever told you that you had kidney disease (e.g. nephritis)?”, meaning it may represent a range of mild to severe kidney disease, making interpretation of this result more challenging. There was no evidence that any of the other comorbidities we assessed, including arthritis, diabetes or cancer, increased susceptibility to frequent cold sores. Increased sunlight exposure,[21, 27, 28] fatigue[21] and psychological stress[21, 29] have previously been posited as risk factors for HSV-1 reactivation, findings which are supported in this study.

This is one of the largest population-based studies to assess childhood characteristics as risk factors for HSV-1-seropositivity and clinical comorbidities as risk factors for oro-labial HSV reactivation. However, there were limitations. For some of the risk factors of interest, the study was underpowered; this may explain our null findings regarding some of the clinical comorbidities we assessed as risk factors for reactivation. Although the study questions addressed here were derived following data collection, meaning the data would not be subject to recall or reporter bias, those participants with poorer overall health may recollect illness differently to those who are very well, potentially overestimating the associations between HSV-1 reactivation and comorbidities such as kidney disease.

Another limitation is that the participation rate for the original study cohort was 39.2% and although this cohort is similar to the general UK population[8] there is risk of bias from the healthy participant effect, which may have led to an underestimation of both HSV-1 seropositivity and reactivation. Nevertheless, our estimates of seroprevalence concur with previous research, suggesting this has not impacted dramatically on our findings.

The study may also have suffered from misclassification. First, reactivation was self-reported and only measured once during the follow-up of EPIC Norfolk. However, misclassification is likely to be non-differential according to our main exposures of interest (severe immunosuppression and other common comorbidities of mid-late life), resulting in our estimates being reduced toward the null. Additionally, outdoor job was used as a proxy for UV exposure, as there were no data available on sunlight exposure; our results were however in line with previous research. There may also be misclassification of our time-varying risk factors of interest, particularly from using data derived in adulthood to act as a proxy for childhood data, such as socioeconomic status and urban living.

As EPIC data did not specifically ask for genital HSV and serology cannot discriminate genital from non-genital HSV, we were not able to differentiate their specific risk factors, which may differ. However, genital HSV-1 is thought to have been rare prior to the 1980s,[23, 30] with most infection being non-genital and occurring during childhood and we therefore assume that genital HSV-1 is rare in the EPIC Norfolk cohort.

This study has improved our understanding of the characteristics of people who are at high risk of HSV-1 infection and frequent reactivation and will help to inform public health prevention and control strategies. Our estimates provide a baseline figure which can inform future research in this area. However, further studies are needed to understand whether clinical comorbidities increase the risk of HSV-1 reactivation.

These data will inform research into HSV-1 in electronic healthcare data. It is likely there will be significant under-ascertainment of HSV-1 reactivation in primary care data; the degree of under-ascertainment can now be quantified. Our better understanding of the risk factors for seroprevalence and reactivation of HSV-1 will also help us understand some of the potential biases and inform interpretation of the results of future studies.

## Conclusions

In this large population-based study, HSV-1 seropositivity and frequent reactivation in a community dwelling ageing population is common. Lower socioeconomic status, urban living and a greater number of siblings appear to be associated with a greater risk of HSV-1 infection. This study showed very little evidence that common clinical comorbidities increase the risk of orolabial HSV reactivation apart from possibly kidney disease, though the study was underpowered for some exposures. These data will help inform studies of HSV-1.

## Supporting information

S1 Fig Directed acyclic graphs (DAGs) illustrating implicitly assumed causal structure underlying our adjusted models. (DOCX)

S1 Table Baseline characteristics by cohort status. Figures are numbers (percentage) unless otherwise stated.
(DOCX)

S2 Table Determinants of HSV-1 reactivation, stratified by age. (DOCX)

S3 Table Determinants of HSV-1 reactivation, stratified by sex. (DOCX)
