# Supplementary material for: Risk factors for herpes simplex virus type-1 infection and reactivation: Cross-sectional studies among EPIC-Norfolk participants
Source: PLoS One. 2019 May 9;14(5):e0215553. doi: 10.1371/journal.pone.0215553 (PMC6508674; doi:10.1371/journal.pone.0215553)
Supplement: S1 Fig — (DOCX) [file pone.0215553.s001.docx]

S1 Fig. Directed acyclic graphs (DAGs) illustrating implicitly assumed causal structure underlying our adjusted models

Below are two directed acyclic graphs (DAGs). A DAG is a visual representation of the assumptions regarding causal relationships between variables in a study. It allows us to determine which variables must be controlled for, in order to derive the unconfounded effect estimates.

1. Directed acyclic graph (DAG) illustrating implicitly assumed causal structure underlying our adjusted models for study 1, risk factors for HSV-1 infection.


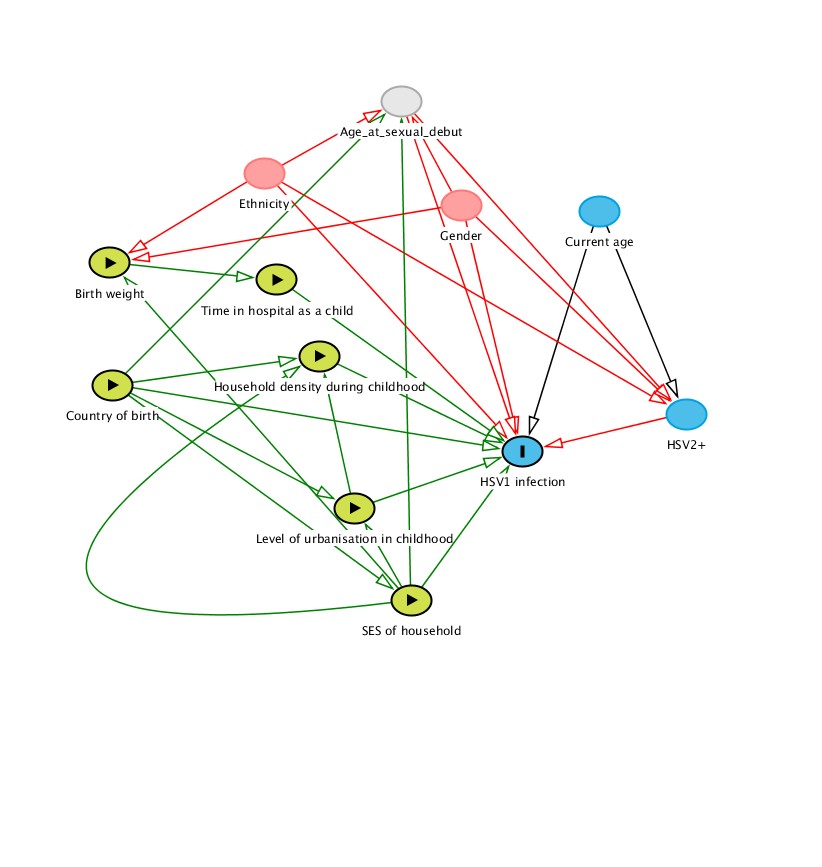

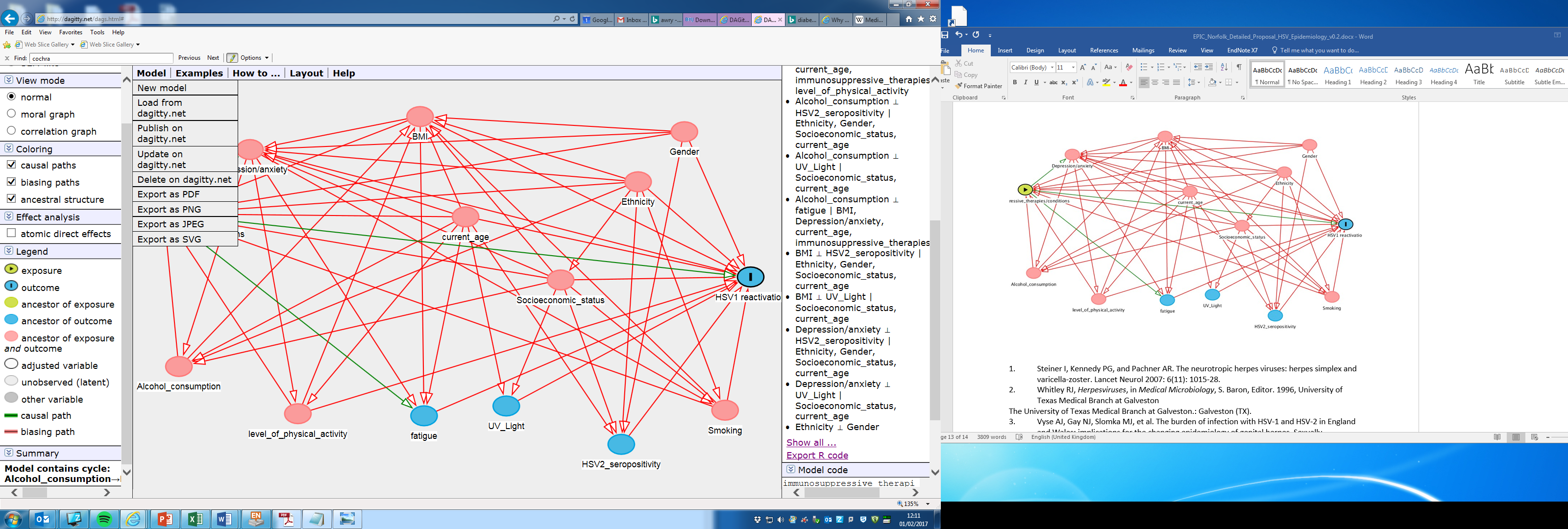

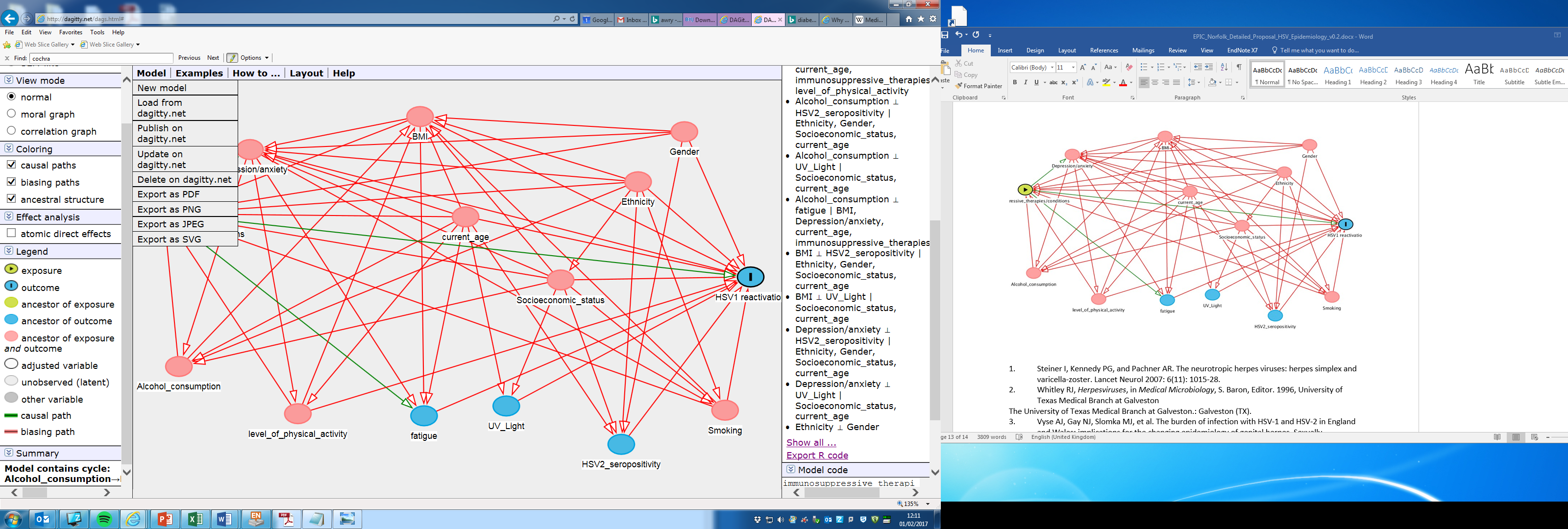


**Legend**

This DAG shows that to measure the total effect of childhood environmental and social conditions such as country of birth, household density at birth and socio-economic status on the risk of HSV-1 infection, we must control for gender and ethnicity.

1. Directed acyclic graph (DAG) illustrating implicitly assumed causal structure underlying our adjusted models for study 2, risk factors for HSV-1 reactivation.


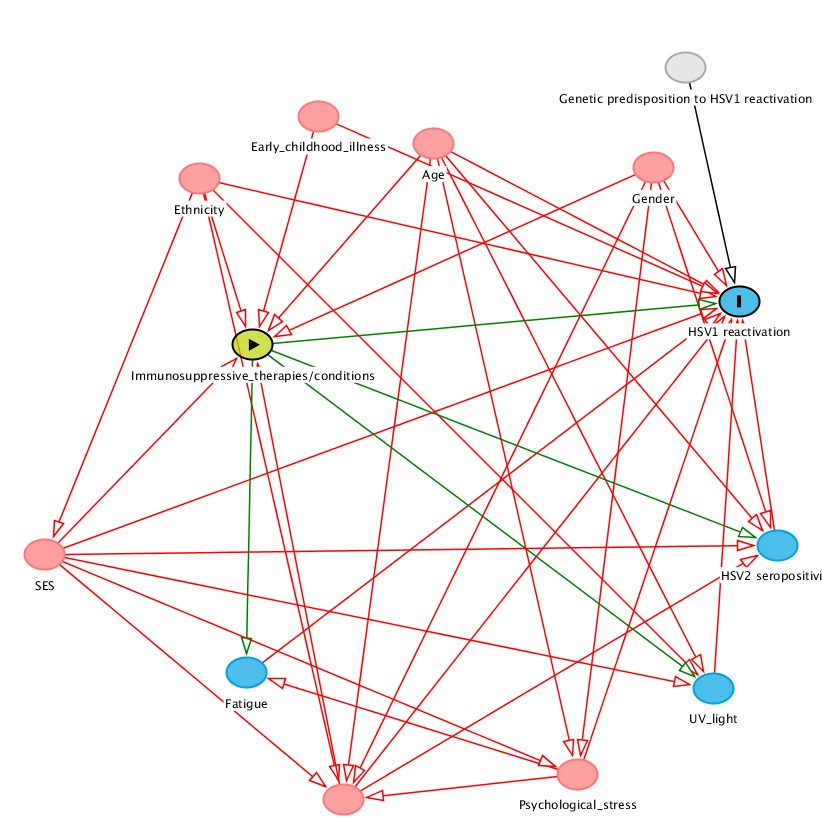

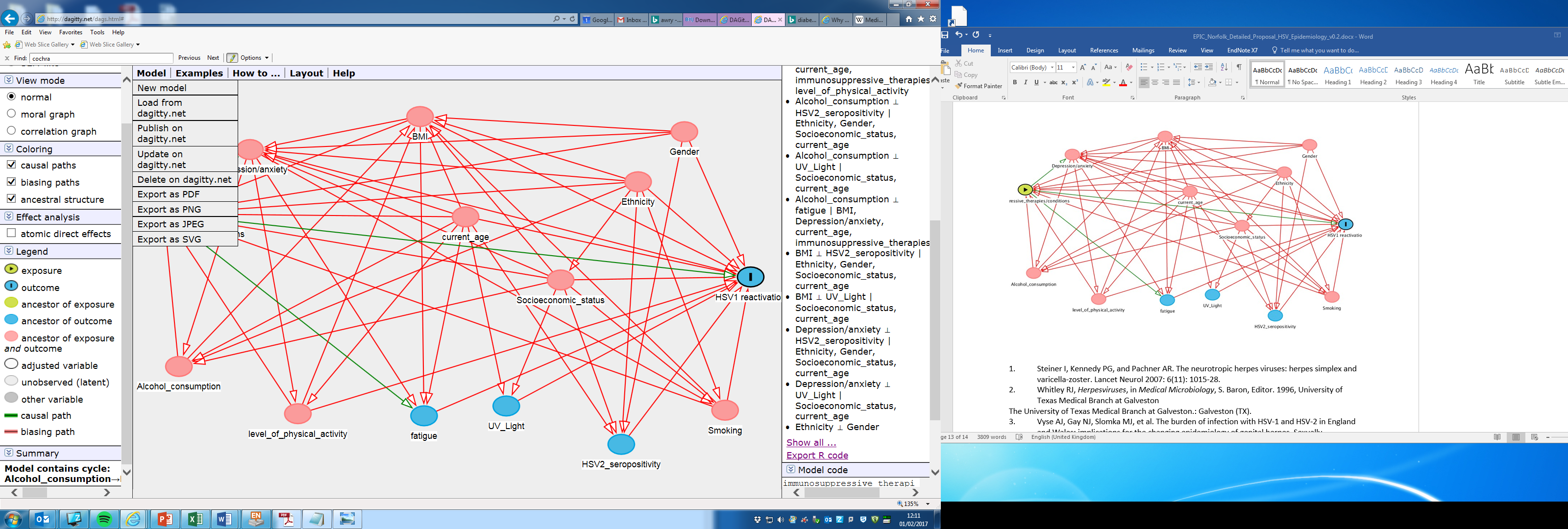

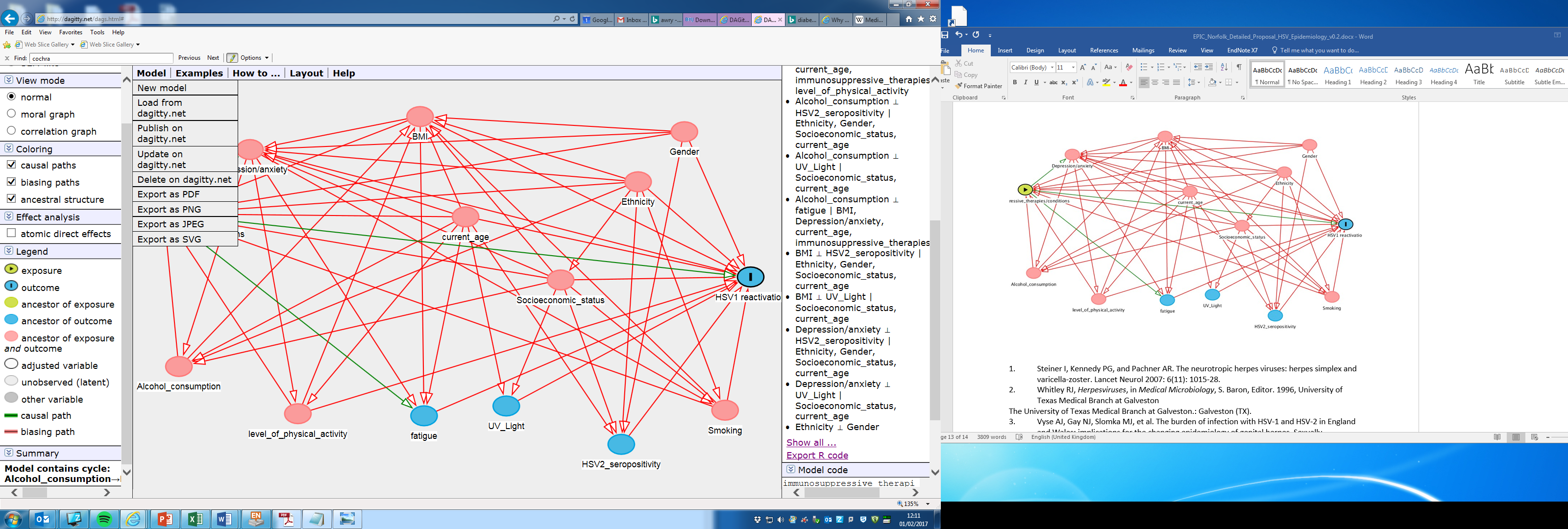


**Legend**

**Note:** Immunosuppressive conditions or therapies include: corticosteroid use, other immunosuppressive medications (for example chemotherapy and methotrexate), cancers (excluding skin cancer), rheumatoid arthritis, ulcerative colitis, Crohn’s disease, kidney disease and diabetes.

The graph shows that to measure the total effect of immunosuppressive conditions and therapies on HSV-1 reactivation, we must control for gender, age, ethnicity, SES, lifestyle risk factors and psychological stress.
