# Supplementary material for: Risk factors for herpes simplex virus type-1 infection and reactivation: Cross-sectional studies among EPIC-Norfolk participants
Source: PLoS One. 2019 May 9;14(5):e0215553. doi: 10.1371/journal.pone.0215553 (PMC6508674; doi:10.1371/journal.pone.0215553)
Supplement: S1 Table — Figures are numbers (percentage) unless otherwise stated. (DOCX) [file pone.0215553.s002.docx]

| **S1 Table. Baseline characteristics by cohort status. Figures are numbers (percentage) unless otherwise stated** | | | | |
| --- | --- | --- | --- | --- |
|  |  |  |  |  |
|  | Recruited  (1993), n (%) | Attended 1HC  (1993-1998), n (%) | First cohort, n (%) | Second cohort, n (%) |
| No of participants | 30445 (100%) | 25639 (100%) | 9929 (100%) | 4934 (100%) |
|  |  |  |  |  |
| Gender |  |  |  |  |
| Females | 16740 (55.0) | 14030 (54.7) | 5882 (59.2) | 2994 (60.7) |
| Males | 13698 (45.0) | 11606 (45.3) | 4047 (40.8) | 1940 (39.3) |
| Missing | 7 ( 0.0) | 3 ( 0.0) | 0 ( 0.0) | 0 ( 0.0) |
|  |  |  |  |  |
| Age (years) |  |  |  |  |
| 40-44 | 1290 ( 4.2) | 1033 ( 4.0) | 403 ( 4.1) | 180 ( 3.6) |
| 45-54 | 10097 (33.2) | 8585 (33.5) | 3338 (33.6) | 1623 (32.9) |
| 55-64 | 9202 (30.2) | 7916 (30.9) | 3356 (33.8) | 1760 (35.7) |
| 65-74 | 8870 (29.1) | 7338 (28.6) | 2609 (26.3) | 1278 (25.9) |
| 75+ | 979 ( 3.2) | 764 ( 3.0) | 223 ( 2.2) | 93 ( 1.9) |
| Missing | 7 ( 0.0) | 3 ( 0.0) | 0 ( 0.0) | 0 ( 0.0) |
|  |  |  |  |  |
| BMI category |  |  |  |  |
| Underweight | - | 124 ( 0.5) | 41 ( 0.4) | 12 ( 0.2) |
| Normal Weight | - | 9945 (38.8) | 4038 (40.7) | 2012 (40.8) |
| Overweight | - | 11599 (45.2) | 4162 (41.9) | 2041 (41.4) |
| Obese | - | 3911 (15.3) | 1220 (12.3) | 628 (12.7) |
| Missing | - | 60 ( 0.2) | 468 ( 4.7) | 241 ( 4.9) |
|  |  |  |  |  |
| Smoking status |  |  |  |  |
| current smoker | 3729 (12.2) | 2983 (11.6) | 864 ( 8.7) | 427 ( 8.7) |
| former smoker | 12703 (41.7) | 10760 (42.0) | 4062 (40.9) | 2100 (42.6) |
| never smoked | 13719 (45.1) | 11673 (45.5) | 4924 (49.6) | 2369 (48.0) |
| Missing | 294 ( 1.0) | 223 ( 0.9) | 79 ( 0.8) | 38 ( 0.8) |
|  |  |  |  |  |
| Self reported high blood pressure at baseline | | |  |  |
| Yes | 4500 (14.8) | 3662 (14.3) | 1265 (12.7) | 615 (12.5) |
| No | 25878 (85.0) | 21931 (85.5) | 8651 (87.1) | 4315 (87.5) |
| Missing | 67 ( 0.2) | 46 ( 0.2) | 13 ( 0.1) | 4 ( 0.1) |
