# Supplementary material for: Risk factors for herpes simplex virus type-1 infection and reactivation: Cross-sectional studies among EPIC-Norfolk participants
Source: PLoS One. 2019 May 9;14(5):e0215553. doi: 10.1371/journal.pone.0215553 (PMC6508674; doi:10.1371/journal.pone.0215553)
Supplement: S2 Table — (DOCX) [file pone.0215553.s003.docx]

| **S2 Table. Determinants of HSV-1 reactivation, stratified by age** | | |  |  |  |  |  |  |
| --- | --- | --- | --- | --- | --- | --- | --- | --- |
|  |  |  |  |  |  |  |  |  |
|  | Age 40-64 years | | | | Age 65-89 years | | | |
|  | Overall n (%) | Prevalence HSV-1 reactivation n (%) | Unadjusted OR (95% CI) | Adjusted* OR (95% CI) | Overall n (%) | Prevalence HSV-1 reactivation n (%) | Unadjusted OR (95% CI) | Adjusted* OR (95% CI) |
| No of participants | 2186 (100%) | 411 (18.8) |  |  | 2748 (100%) | 459(16.7) |  |  |
|  |  |  |  |  |  |  |  |  |
| Level of HSV-1 IgG, in tertiles |  |  |  |  |  |  |  |  |
| Low | 839 (38.4) | 98 (11.7) | 1 | 1 | 805 (29.3) | 94 (11.7) | 1 | 1 |
| Medium | 703 (32.2) | 165 (23.5) | 2.32 (1.76-3.05) | 2.32 (1.72-3.12) | 942 (34.3) | 177 (18.8) | 1.75 (1.34-2.29) | 1.92 (1.42-2.58) |
| High | 644 (29.5) | 148 (23.0) | 2.26 (1.71-2.98) | 2.31 (1.70-3.13) | 1001 (36.4) | 188 (18.8) | 1.75 (1.34-2.28) | 1.95 (1.45-2.62) |
| Missing | 33 (0.3) |  |  |  | 33 (0.3) |  |  |  |
|  |  |  |  |  |  |  |  |  |
| Demographic characteristics |  |  |  |  |  |  |  |  |
|  |  |  |  |  |  |  |  |  |
| Gender (1HC) |  |  |  |  |  |  |  |  |
| Males | 744 (34.0) | 129 (17.3) | 1 | 1 | 1196 (43.5) | 192 (16.1) | 1 | 1 |
| Females | 1442 (66.0) | 282 (19.6) | 1.16 (0.92-1.46) | 1.09 (0.85-1.41) | 1552 (56.5) | 267 (17.2) | 1.09 (0.89-1.33) | 1.06 (0.84-1.34) |
|  |  |  |  |  |  |  |  |  |
| Ethnicity (1HC) |  |  |  |  |  |  |  |  |
| White | 2168 (99.2) | 409 (18.9) | 1 | 1 | 2731 (99.4) | 457 (16.7) | 1 | 1 |
| Other | 18 ( 0.8) | 2 (11.1) | 0.54 (0.12-2.35) | 0.63 (0.08-5.21) | 17 ( 0.6) | 2 (11.8) | 0.66 (0.15-2.91) | 0.85 (0.19-3.89) |
|  |  |  |  |  |  |  |  |  |
| Education level (1HC) |  |  |  |  |  |  |  |  |
| None | 588 (26.9) | 109 (18.5) | 1 | 1 | 1134 (41.3) | 187 (16.5) | 1 | 1 |
| O-Level | 300 (13.7) | 59 (19.7) | 1.08 (0.76-1.53) | 0.98 (0.66-1.44) | 247 ( 9.0) | 51 (20.6) | 1.32 (0.93-1.86) | 1.17 (0.81-1.71) |
| A-level | 936 (42.8) | 172 (18.4) | 0.99 (0.76-1.29) | 0.98 (0.73-1.32) | 1091 (39.7) | 183 (16.8) | 1.02 (0.82-1.28) | 0.96 (0.75-1.23) |
| Degree or higher | 362 (16.6) | 71 (19.6) | 1.07 (0.77-1.50) | 0.97 (0.67-1.41) | 274 (10.0) | 38 (13.9) | 0.82 (0.56-1.19) | 0.83 (0.56-1.23) |
| Missing | 2 (0.04) |  |  |  | 2 (0.04) |  |  |  |
|  |  |  |  |  |  |  |  |  |
| Townsend quintile (1HC)¹ |  |  |  |  |  |  |  |  |
| Q1 (most affluent) | 1232 (56.4) | 240 (19.5) | 1 | 1 | 1592 (57.9) | 264 (16.6) | 1 | 1 |
| Q2 | 567 (25.9) | 103 (18.2) | 0.92 (0.71-1.19) | 0.90 (0.68-1.19) | 679 (24.7) | 117 (17.2) | 1.05 (0.82-1.33) | 1.03 (0.80-1.34) |
| Q3 | 247 (11.3) | 39 (15.8) | 0.78 (0.54-1.12) | 0.76 (0.51-1.14) | 283 (10.3) | 41 (14.5) | 0.85 (0.60-1.22) | 0.84 (0.57-1.24) |
| Q4 | 112 ( 5.1) | 22 (19.6) | 1.01 (0.62-1.64) | 1.07 (0.64-1.81) | 166 ( 6.0) | 36 (21.7) | 1.39 (0.94-2.06) | 1.45 (0.94-2.23) |
| Q5 (most deprived) | 21 ( 1.0) | 6 (28.6) | 1.65 (0.63-4.31) | 2.28 (0.83-6.30) | 22 ( 0.8) | 1 ( 4.5) | 0.24 (0.03-1.79) | 0.33 (0.04-2.49) |
| Missing | 13 (0.3) |  |  |  | 13 (0.3) |  |  |  |
|  |  |  |  |  |  |  |  |  |
| Immunosuppressive medications and conditions | |  |  |  |  |  |  |  |
|  |  |  |  |  |  |  |  |  |
| Corticosteroids (Follow3) |  |  |  |  |  |  |  |  |
| no | 2013 (92.1) | 379 (18.8) | 1 | 1 | 2294 (83.5) | 392 (17.1) | 1 | 1 |
| yes | 59 ( 2.7) | 16 (27.1) | 1.60 (0.89-2.88) | 1.37 (0.71-2.62) | 134 ( 4.9) | 18 (13.4) | 0.75 (0.45-1.25) | 0.73 (0.42-1.26) |
| Missing | 434 (8.8) |  |  |  | 434 (8.8) |  |  |  |
|  |  |  |  |  |  |  |  |  |
| Other immunosuppressive medications (Follow3) | |  |  |  |  |  |  |  |
| no | 2164 (99.0) | 407 (18.8) | 1 | 1 | 2711 (98.7) | 453 (16.7) | 1 | 1 |
| yes | 22 ( 1.0) | 4 (18.2) | 0.96 (0.32-2.85) | 1.17 (0.38-3.60) | 37 ( 1.3) | 6 (16.2) | 0.96 (0.40-2.33) | 0.68 (0.23-1.96) |
| Missing | 0 (0) |  |  |  | 0 (0) |  |  |  |
|  |  |  |  |  |  |  |  |  |
| Non-steroidal anti-inflammatories (Follow3) |  |  |  |  |  |  |  |  |
| no | 1777 (81.3) | 323 (18.2) | 1 | 1 | 1906 (69.4) | 340 (17.8) | 1 | 1 |
| yes | 409 (18.7) | 88 (21.5) | 1.23 (0.95-1.61) | 1.35 (1.01-1.80) | 842 (30.6) | 119 (14.1) | 0.76 (0.60-0.95) | 0.74 (0.57-0.94) |
| Missing | 0 (0) |  |  |  | 0 (0) |  |  |  |
|  |  |  |  |  |  |  |  |  |
| Arthritis (2HC) |  |  |  |  |  |  |  |  |
| no | 1614 (73.8) | 299 (18.5) | 1 | 1 | 1412 (51.4) | 224 (15.9) | 1 | 1 |
| yes | 415 (19.0) | 82 (19.8) | 1.08 (0.82-1.42) | 1.08 (0.80-1.47) | 1094 (39.8) | 191 (17.5) | 1.12 (0.91-1.39) | 1.20 (0.94-1.52) |
| Missing | 399 (8.1) |  |  |  | 399 (8.1) |  |  |  |
|  |  |  |  |  |  |  |  |  |
| Ulcerative Colitis / Crohn`s Disease (Follow3) |  |  |  |  |  |  |  |  |
| no | 2066 (94.5) | 390 (18.9) | 1 | 1 | 2474 (90.0) | 420 (17.0) | 1 | 1 |
| yes | 36 ( 1.6) | 7 (19.4) | 1.04 (0.45-2.39) | 0.95 (0.38-2.39) | 39 ( 1.4) | 7 (17.9) | 1.07 (0.47-2.44) | 1.08 (0.45-2.57) |
| Missing | 319 (6.5) |  |  |  | 319 (6.5) |  |  |  |
|  |  |  |  |  |  |  |  |  |
| Kidney Disease (Follow3) |  |  |  |  |  |  |  |  |
| no | 2076 (95.0) | 391 (18.8) | 1 | 1 | 2503 (91.1) | 421 (16.8) | 1 | 1 |
| yes | 30 ( 1.4) | 7 (23.3) | 1.31 (0.56-3.08) | 1.35 (0.56-3.23) | 31 ( 1.1) | 10 (32.3) | 2.35 (1.10-5.04) | 2.66 (1.14-6.21) |
| Missing | 294 (6.0) |  |  |  | 294 (6.0) |  |  |  |
|  |  |  |  |  |  |  |  |  |
| Diabetes (2HC) |  |  |  |  |  |  |  |  |
| no | 2149 (98.3) | 403 (18.8) | 1 | 1 | 2600 (94.6) | 434 (16.7) | 1 | 1 |
| yes | 37 ( 1.7) | 8 (21.6) | 1.20 (0.54-2.63) | 0.89 (0.34-2.36) | 148 ( 5.4) | 25 (16.9) | 1.01 (0.65-1.58) | 0.93 (0.57-1.54) |
|  |  |  |  |  |  |  |  |  |
| Cancer (from cancer registry data) |  |  |  |  |  |  |  |  |
| 0 | 2019 (92.4) | 388 (19.2) | 1 | 1 | 2397 (87.2) | 400 (16.7) | 1 | 1 |
| 1 | 167 ( 7.6) | 23 (13.8) | 0.67 (0.43-1.06) | 0.62 (0.37-1.02) | 351 (12.8) | 59 (16.8) | 1.01 (0.75-1.36) | 0.99 (0.71-1.38) |
|  |  |  |  |  |  |  |  |  |
| Other known risk factors for HSV reactivation |  |  |  |  |  |  |  |  |
|  |  |  |  |  |  |  |  |  |
| UV light exposure: had an outdoor job (1HC) |  |  |  |  |  |  |  |  |
| No | 1891 (86.5) | 347 (18.4) | 1 | 1 | 2311 (84.1) | 373 (16.1) | 1 | 1 |
| Yes | 295 (13.5) | 64 (21.7) | 1.23 (0.91-1.66) | 1.34 (0.94-1.91) | 434 (15.8) | 85 (19.6) | 1.27 (0.97-1.64) | 1.33 (0.99-1.79) |
| Missing | 3 (0.1) |  |  |  | 3 (0.1) |  |  |  |
|  |  |  |  |  |  |  |  |  |
| Concentration of 25-Hydroxyvitamin D3 (nmol/L)(2HC) | |  |  |  |  |  |  |  |
| Deficient (0-29) | 205 ( 9.4) | 32 (15.6) | 0.81 (0.53-1.23) | 0.86 (0.54-1.37) | 302 (11.0) | 41 (13.6) | 0.74 (0.51-1.07) | 0.73 (0.48-1.10) |
| Insufficiency (30-49) | 609 (27.9) | 109 (17.9) | 0.95 (0.72-1.26) | 1.02 (0.75-1.39) | 883 (32.1) | 145 (16.4) | 0.92 (0.72-1.18) | 0.98 (0.75-1.29) |
| Adequate (50-69) | 715 (32.7) | 133 (18.6) | 1 | 1 | 887 (32.3) | 156 (17.6) | 1 | 1 |
| High (70-89) | 422 (19.3) | 90 (21.3) | 1.19 (0.88-1.60) | 1.24 (0.89-1.72) | 465 (16.9) | 87 (18.7) | 1.08 (0.81-1.44) | 1.11 (0.81-1.53) |
| Undesirably high (90+) | 228 (10.4) | 45 (19.7) | 1.08 (0.74-1.57) | 1.26 (0.84-1.89) | 200 ( 7.3) | 28 (14.0) | 0.76 (0.49-1.18) | 0.73 (0.45-1.20) |
| Missing | 18 (0.4) |  |  |  | 18 (0.4) |  |  |  |
|  |  |  |  |  |  |  |  |  |
| Do you feel tired? (Follow3) |  |  |  |  |  |  |  |  |
| All of the time | 50 ( 2.3) | 16 (32.0) | 1.97 (1.06-3.66) | 1.75 (0.86-3.58) | 62 ( 2.3) | 11 (17.7) | 1.11 (0.57-2.17) | 0.96 (0.43-2.11) |
| Most of the time | 121 ( 5.5) | 34 (28.1) | 1.64 (1.06-2.52) | 1.54 (0.96-2.48) | 137 ( 5.0) | 37 (27.0) | 1.91 (1.27-2.86) | 1.72 (1.09-2.69) |
| A good bit of the time | 288 (13.2) | 54 (18.8) | 0.97 (0.69-1.36) | 0.82 (0.56-1.20) | 263 ( 9.6) | 53 (20.2) | 1.30 (0.93-1.82) | 1.24 (0.86-1.79) |
| Some of the time | 846 (38.7) | 163 (19.3) | 1 | 1 | 1206 (43.9) | 196 (16.3) | 1 | 1 |
| A little of the time | 763 (34.9) | 134 (17.6) | 0.89 (0.69-1.15) | 0.93 (0.71-1.22) | 854 (31.1) | 138 (16.2) | 0.99 (0.78-1.26) | 0.98 (0.76-1.28) |
| None of the time | 112 ( 5.1) | 9 ( 8.0) | 0.37 (0.18-0.74) | 0.30 (0.13-0.71) | 182 ( 6.6) | 21 (11.5) | 0.67 (0.42-1.09) | 0.73 (0.44-1.21) |
| Missing | 50 (1.0) |  |  |  | 50 (1.0) |  |  |  |
|  |  |  |  |  |  |  |  |  |
| Has stress affected your health? (1HC) |  |  |  |  |  |  |  |  |
| Not at all | 512 (23.4) | 74 (14.5) | 1 | 1 | 844 (30.7) | 131 (15.5) | 1 | 1 |
| A little | 889 (40.7) | 170 (19.1) | 1.40 (1.04-1.88) | 1.40 (1.04-1.90) | 980 (35.7) | 168 (17.1) | 1.13 (0.88-1.45) | 1.10 (0.85-1.42) |
| A moderate amount | 366 (16.7) | 78 (21.3) | 1.60 (1.13-2.28) | 1.59 (1.12-2.27) | 381 (13.9) | 72 (18.9) | 1.27 (0.92-1.74) | 1.26 (0.91-1.73) |
| A great deal | 174 ( 8.0) | 38 (21.8) | 1.65 (1.07-2.56) | 1.70 (1.09-2.64) | 189 ( 6.9) | 38 (20.1) | 1.37 (0.92-2.05) | 1.27 (0.84-1.91) |
| Missing | 599 (12.1) |  |  |  | 599 (12.1) |  |  |  |
|  |  |  |  |  |  |  |  |  |
| Health behaviours and anthropometry |  |  |  |  |  |  |  |  |
|  |  |  |  |  |  |  |  |  |
| BMI category (2HC) |  |  |  |  |  |  |  |  |
| Underweight | 8 ( 0.4) | 2 (25.0) | 1.36 (0.27-6.81) | 1.04 (0.11-9.55) | 5 ( 0.2) | 1 (20.0) | 1.19 (0.13-10.76) | 1.14 (0.12-10.64) |
| Normal Weight | 911 (41.7) | 179 (19.6) | 1 | 1 | 861 (31.3) | 149 (17.3) | 1 | 1 |
| Overweight | 937 (42.9) | 168 (17.9) | 0.89 (0.71-1.13) | 0.87 (0.67-1.12) | 1380 (50.2) | 220 (15.9) | 0.91 (0.72-1.14) | 0.87 (0.68-1.12) |
| Obese | 325 (14.9) | 62 (19.1) | 0.96 (0.70-1.33) | 0.85 (0.60-1.22) | 501 (18.2) | 89 (17.8) | 1.03 (0.77-1.38) | 1.09 (0.80-1.49) |
| Missing | 6 (0.1) |  |  |  | 6 (0.1) |  |  |  |
|  |  |  |  |  |  |  |  |  |
| Smoking status (Follow3) |  |  |  |  |  |  |  |  |
| current smoker | 223 (10.2) | 37 (16.6) | 0.80 (0.54-1.17) | 0.74 (0.48-1.14) | 148 ( 5.4) | 13 ( 8.8) | 0.46 (0.26-0.84) | 0.39 (0.20-0.76) |
| former smoker | 900 (41.2) | 162 (18.0) | 0.88 (0.70-1.10) | 0.90 (0.70-1.15) | 1393 (50.7) | 237 (17.0) | 0.99 (0.80-1.21) | 0.97 (0.77-1.22) |
| never smoked | 1057 (48.4) | 211 (20.0) | 1 | 1 | 1187 (43.2) | 204 (17.2) | 1 | 1 |
| Missing | 26 (0.5) |  |  |  | 26 (0.5) |  |  |  |
|  |  |  |  |  |  |  |  |  |
| HSV-2 infection |  |  |  |  |  |  |  |  |
| 0 | 2048 (93.7) | 395 (19.3) | 1 | 1 | 2623 (95.5) | 440 (16.8) | 1 | 1 |
| 1 | 138 ( 6.3) | 16 (11.6) | 0.55 (0.32-0.93) | 0.53 (0.30-0.94) | 125 ( 4.5) | 19 (15.2) | 0.89 (0.54-1.46) | 0.91 (0.53-1.56) |
| *Adjusted for gender, age, ethnicity, SES, lifestyle factors (smoking and BMI) and psychological stress**Additionally adjusted for UV light exposure, fatigue and HSV-2 status | | | | | | | |  |
