# Supplementary material for: Risk factors for herpes simplex virus type-1 infection and reactivation: Cross-sectional studies among EPIC-Norfolk participants
Source: PLoS One. 2019 May 9;14(5):e0215553. doi: 10.1371/journal.pone.0215553 (PMC6508674; doi:10.1371/journal.pone.0215553)
Supplement: S3 Table — (DOCX) [file pone.0215553.s004.docx]

| **S3 Table. Determinants of HSV-1 reactivation, stratified by sex** | | |  |  |  |  |  |  |
| --- | --- | --- | --- | --- | --- | --- | --- | --- |
|  |  |  |  |  |  |  |  |  |
|  | Men | | | | Women | | | |
|  | Overall n (%) | Prevalence HSV-1 reactivation n (%) | Unadjusted OR (95% CI) | Adjusted* OR (95% CI) | Overall n (%) | Prevalence HSV-1 reactivation n (%) | Unadjusted OR (95% CI) | Adjusted* OR (95% CI) |
| No of participants | 1940 (100%) | 321 (16.5) |  |  | 2994 (100%) | 549 (18.3) |  |  |
|  |  |  |  |  |  |  |  |  |
| Level of HSV-1 IgG, in tertiles |  |  |  |  |  |  |  |  |
| Low | 617 (31.8) | 71 (11.5) | 1 | 1 | 1027 (34.3) | 121 (11.8) | 1 | 1 |
| Medium | 610 (31.4) | 111 (18.2) | 1.71 (1.24-2.36) | 1.90 (1.33-2.71) | 1035 (34.6) | 231 (22.3) | 2.15 (1.69-2.73) | 2.24 (1.73-2.91) |
| High | 713 (36.8) | 139 (19.5) | 1.86 (1.37-2.54) | 2.08 (1.48-2.93) | 932 (31.1) | 197 (21.1) | 2.01 (1.57-2.57) | 2.12 (1.62-2.77) |
| Missing | 33 (0.3) |  |  |  | 33 (0.3) |  |  |  |
|  |  |  |  |  |  |  |  |  |
| Age in years (3H&LQ) |  |  |  |  |  |  |  |  |
| 40-49 | 26 ( 1.3) | 3 (11.5) | 1 | 1 | 86 ( 2.9) | 16 (18.6) | 1 | 1 |
| 50-59 | 380 (19.6) | 70 (18.4) | 1.73 (0.51-5.93) | 1.34 (0.38-4.74) | 784 (26.2) | 168 (21.4) | 1.19 (0.68-2.11) | 1.14 (0.61-2.10) |
| 60-69 | 667 (34.4) | 111 (16.6) | 1.53 (0.45-5.19) | 1.23 (0.35-4.29) | 1033 (34.5) | 187 (18.1) | 0.97 (0.55-1.70) | 0.95 (0.52-1.76) |
| 70-79 | 679 (35.0) | 110 (16.2) | 1.48 (0.44-5.02) | 1.18 (0.34-4.11) | 871 (29.1) | 151 (17.3) | 0.92 (0.52-1.62) | 0.99 (0.53-1.83) |
| 80-89 | 185 ( 9.5) | 26 (14.1) | 1.25 (0.35-4.48) | 0.94 (0.25-3.49) | 212 ( 7.1) | 24 (11.3) | 0.56 (0.28-1.11) | 0.51 (0.24-1.08) |
|  |  |  |  |  |  |  |  |  |
| Demographic characteristics |  |  |  |  |  |  |  |  |
|  |  |  |  |  |  |  |  |  |
| Ethnicity (1HC) |  |  |  |  |  |  |  |  |
| White | 1922 (99.1) | 319 (16.6) | 1 | 1 | 2977 (99.4) | 547 (18.4) | 1 | 1 |
| Other | 18 ( 0.9) | 2 (11.1) | 0.63 (0.14-2.75) | 0.85 (0.18-3.97) | 17 ( 0.6) | 2 (11.8) | 0.59 (0.14-2.60) | 0.52 (0.06-4.24) |
|  |  |  |  |  |  |  |  |  |
| Education level (1HC) |  |  |  |  |  |  |  |  |
| None | 536 (27.6) | 89 (16.6) | 1 | 1 | 1186 (39.6) | 207 (17.5) | 1 | 1 |
| O-Level | 162 ( 8.4) | 30 (18.5) | 1.14 (0.72-1.80) | 1.20 (0.74-1.95) | 385 (12.9) | 80 (20.8) | 1.24 (0.93-1.65) | 1.04 (0.75-1.43) |
| A-level | 931 (48.0) | 153 (16.4) | 0.99 (0.74-1.31) | 0.96 (0.70-1.31) | 1096 (36.6) | 202 (18.4) | 1.07 (0.86-1.32) | 0.98 (0.78-1.24) |
| Degree or higher | 310 (16.0) | 49 (15.8) | 0.94 (0.64-1.38) | 0.94 (0.62-1.42) | 326 (10.9) | 60 (18.4) | 1.07 (0.78-1.47) | 0.88 (0.62-1.24) |
| Missing | 2 (0.04) |  |  |  | 2 (0.04) |  |  |  |
|  |  |  |  |  |  |  |  |  |
| Townsend quintile (1HC)¹ |  |  |  |  |  |  |  |  |
| Q1 (most affluent) | 1145 (59.0) | 202 (17.6) | 1 | 1 | 1679 (56.1) | 302 (18.0) | 1 | 1 |
| Q2 | 493 (25.4) | 74 (15.0) | 0.82 (0.62-1.10) | 0.73 (0.53-1.01) | 753 (25.2) | 146 (19.4) | 1.10 (0.88-1.37) | 1.14 (0.90-1.44) |
| Q3 | 197 (10.2) | 23 (11.7) | 0.62 (0.39-0.98) | 0.49 (0.29-0.84) | 333 (11.1) | 57 (17.1) | 0.94 (0.69-1.29) | 1.03 (0.74-1.44) |
| Q4 | 84 ( 4.3) | 18 (21.4) | 1.27 (0.74-2.19) | 1.52 (0.85-2.72) | 194 ( 6.5) | 40 (20.6) | 1.18 (0.82-1.71) | 1.21 (0.80-1.81) |
| Q5 (most deprived) | 16 ( 0.8) | 4 (25.0) | 1.56 (0.50-4.87) | 1.73 (0.53-5.65) | 27 ( 0.9) | 3 (11.1) | 0.57 (0.17-1.91) | 0.97 (0.28-3.38) |
| Missing | 13 (0.3) |  |  |  | 13 (0.3) |  |  |  |
|  |  |  |  |  |  |  |  |  |
| Immunosuppressive medications and conditions | |  |  |  |  |  |  |  |
|  |  |  |  |  |  |  |  |  |
| Corticosteroids (Follow3) |  |  |  |  |  |  |  |  |
| no | 1663 (85.7) | 279 (16.8) | 1 | 1 | 2644 (88.3) | 492 (18.6) | 1 | 1 |
| yes | 70 ( 3.6) | 8 (11.4) | 0.64 (0.30-1.35) | 0.63 (0.28-1.40) | 123 ( 4.1) | 26 (21.1) | 1.17 (0.75-1.83) | 1.11 (0.68-1.82) |
| Missing | 434 (8.8) |  |  |  | 434 (8.8) |  |  |  |
|  |  |  |  |  |  |  |  |  |
| Other immunosuppressive medications (Follow3) | |  |  |  |  |  |  |  |
| no | 1918 (98.9) | 319 (16.6) | 1 | 1 | 2957 (98.8) | 541 (18.3) | 1 | 1 |
| yes | 22 ( 1.1) | 2 ( 9.1) | 0.50 (0.12-2.16) | 0.57 (0.13-2.52) | 37 ( 1.2) | 8 (21.6) | 1.23 (0.56-2.71) | 1.06 (0.43-2.62) |
| Missing | 0 (0) |  |  |  | 0 (0) |  |  |  |
|  |  |  |  |  |  |  |  |  |
| Non-steroidal anti-inflammatories (Follow3) |  |  |  |  |  |  |  |  |
| no | 1363 (70.3) | 242 (17.8) | 1 | 1 | 2320 (77.5) | 421 (18.1) | 1 | 1 |
| yes | 577 (29.7) | 79 (13.7) | 0.73 (0.56-0.97) | 0.79 (0.58-1.06) | 674 (22.5) | 128 (19.0) | 1.06 (0.85-1.32) | 1.06 (0.83-1.35) |
| Missing | 0 (0) |  |  |  | 0 (0) |  |  |  |
|  |  |  |  |  |  |  |  |  |
| Arthritis (2HC) |  |  |  |  |  |  |  |  |
| no | 1316 (67.8) | 217 (16.5) | 1 | 1 | 1710 (57.1) | 306 (17.9) | 1 | 1 |
| yes | 494 (25.5) | 80 (16.2) | 0.98 (0.74-1.30) | 1.16 (0.85-1.57) | 1015 (33.9) | 193 (19.0) | 1.08 (0.88-1.32) | 1.17 (0.92-1.47) |
| Missing | 399 (8.1) |  |  |  | 399 (8.1) |  |  |  |
|  |  |  |  |  |  |  |  |  |
| Ulcerative Colitis / Crohn`s Disease (Follow3) |  |  |  |  |  |  |  |  |
| no | 1802 (92.9) | 298 (16.5) | 1 | 1 | 2738 (91.4) | 512 (18.7) | 1 | 1 |
| yes | 27 ( 1.4) | 5 (18.5) | 1.15 (0.43-3.05) | 0.88 (0.29-2.72) | 48 ( 1.6) | 9 (18.8) | 1.00 (0.48-2.08) | 1.00 (0.47-2.14) |
| Missing | 319 (6.5) |  |  |  | 319 (6.5) |  |  |  |
|  |  |  |  |  |  |  |  |  |
| Kidney Disease (Follow3) |  |  |  |  |  |  |  |  |
| no | 1819 (93.8) | 302 (16.6) | 1 | 1 | 2760 (92.2) | 510 (18.5) | 1 | 1 |
| yes | 18 ( 0.9) | 2 (11.1) | 0.63 (0.14-2.75) | 0.71 (0.16-3.24) | 43 ( 1.4) | 15 (34.9) | 2.36 (1.25-4.46) | 2.43 (1.23-4.81) |
| Missing | 294 (6.0) |  |  |  | 294 (6.0) |  |  |  |
|  |  |  |  |  |  |  |  |  |
| Diabetes (2HC) |  |  |  |  |  |  |  |  |
| no | 1852 (95.5) | 303 (16.4) | 1 | 1 | 2897 (96.8) | 534 (18.4) | 1 | 1 |
| yes | 88 ( 4.5) | 18 (20.5) | 1.31 (0.77-2.24) | 1.02 (0.54-1.94) | 97 ( 3.2) | 15 (15.5) | 0.81 (0.46-1.41) | 0.82 (0.44-1.52) |
|  |  |  |  |  |  |  |  |  |
| Cancer (from cancer registry data) |  |  |  |  |  |  |  |  |
| 0 | 1781 (91.8) | 300 (16.8) | 1 | 1 | 2635 (88.0) | 488 (18.5) | 1 | 1 |
| 1 | 159 ( 8.2) | 21 (13.2) | 0.75 (0.47-1.21) | 0.66 (0.38-1.14) | 359 (12.0) | 61 (17.0) | 0.90 (0.67-1.21) | 0.95 (0.69-1.31) |
|  |  |  |  |  |  |  |  |  |
| Other known risk factors for HSV reactivation |  |  |  |  |  |  |  |  |
|  |  |  |  |  |  |  |  |  |
| UV light exposure: had an outdoor job (1HC) |  |  |  |  |  |  |  |  |
| No | 1428 (73.6) | 215 (15.1) | 1 | 1 | 2774 (92.7) | 505 (18.2) | 1 | 1 |
| Yes | 511 (26.3) | 106 (20.7) | 1.48 (1.14-1.91) | 1.54 (1.16-2.06) | 218 ( 7.3) | 43 (19.7) | 1.10 (0.78-1.56) | 1.05 (0.71-1.57) |
| Missing | 3 (0.1) |  |  |  | 3 (0.1) |  |  |  |
|  |  |  |  |  |  |  |  |  |
| Concentration of 25-Hydroxyvitamin D3 (nmol/L)(2HC) | |  |  |  |  |  |  |  |
| Deficient (0-29) | 150 ( 7.7) | 16 (10.7) | 0.60 (0.34-1.05) | 0.60 (0.32-1.12) | 357 (11.9) | 57 (16.0) | 0.81 (0.58-1.12) | 0.87 (0.60-1.24) |
| Insufficiency (30-49) | 559 (28.8) | 86 (15.4) | 0.91 (0.67-1.24) | 1.00 (0.72-1.41) | 933 (31.2) | 168 (18.0) | 0.94 (0.74-1.18) | 1.05 (0.81-1.35) |
| Adequate (50-69) | 644 (33.2) | 107 (16.6) | 1 | 1 | 958 (32.0) | 182 (19.0) | 1 | 1 |
| High (70-89) | 382 (19.7) | 74 (19.4) | 1.21 (0.87-1.67) | 1.26 (0.88-1.81) | 505 (16.9) | 103 (20.4) | 1.09 (0.83-1.43) | 1.13 (0.84-1.52) |
| Undesirably high (90+) | 199 (10.3) | 37 (18.6) | 1.15 (0.76-1.73) | 1.15 (0.73-1.81) | 229 ( 7.6) | 36 (15.7) | 0.80 (0.54-1.18) | 0.82 (0.54-1.25) |
| Missing | 18 (0.4) |  |  |  | 18 (0.4) |  |  |  |
|  |  |  |  |  |  |  |  |  |
| Do you feel tired? (Follow3) |  |  |  |  |  |  |  |  |
| All of the time | 29 ( 1.5) | 4 (13.8) | 0.90 (0.31-2.64) | 1.18 (0.39-3.63) | 83 ( 2.8) | 23 (27.7) | 1.64 (0.99-2.70) | 1.39 (0.76-2.52) |
| Most of the time | 69 ( 3.6) | 15 (21.7) | 1.57 (0.86-2.87) | 1.26 (0.62-2.55) | 189 ( 6.3) | 56 (29.6) | 1.80 (1.28-2.53) | 1.75 (1.20-2.54) |
| A good bit of the time | 201 (10.4) | 31 (15.4) | 1.03 (0.67-1.58) | 1.06 (0.67-1.69) | 350 (11.7) | 76 (21.7) | 1.18 (0.89-1.58) | 1.00 (0.73-1.38) |
| Some of the time | 783 (40.4) | 118 (15.1) | 1 | 1 | 1269 (42.4) | 241 (19.0) | 1 | 1 |
| A little of the time | 692 (35.7) | 134 (19.4) | 1.35 (1.03-1.78) | 1.42 (1.05-1.91) | 925 (30.9) | 138 (14.9) | 0.75 (0.59-0.94) | 0.72 (0.57-0.93) |
| None of the time | 146 ( 7.5) | 17 (11.6) | 0.74 (0.43-1.28) | 0.79 (0.44-1.42) | 148 ( 4.9) | 13 ( 8.8) | 0.41 (0.23-0.74) | 0.41 (0.22-0.79) |
| Missing | 50 (1.0) |  |  |  | 50 (1.0) |  |  |  |
|  |  |  |  |  |  |  |  |  |
| Has stress affected your health? (1HC) |  |  |  |  |  |  |  |  |
| Not at all | 567 (29.2) | 88 (15.5) | 1 | 1 | 789 (26.4) | 117 (14.8) | 1 | 1 |
| A little | 746 (38.5) | 121 (16.2) | 1.05 (0.78-1.42) | 1.03 (0.76-1.40) | 1123 (37.5) | 217 (19.3) | 1.38 (1.08-1.76) | 1.37 (1.07-1.76) |
| A moderate amount | 284 (14.6) | 47 (16.5) | 1.08 (0.73-1.59) | 1.07 (0.72-1.58) | 463 (15.5) | 103 (22.2) | 1.64 (1.22-2.21) | 1.64 (1.21-2.21) |
| A great deal | 114 ( 5.9) | 25 (21.9) | 1.53 (0.93-2.52) | 1.53 (0.92-2.55) | 249 ( 8.3) | 51 (20.5) | 1.48 (1.03-2.13) | 1.46 (1.01-2.12) |
| Missing | 599 (12.1) |  |  |  | 599 (12.1) |  |  |  |
|  |  |  |  |  |  |  |  |  |
| Health behaviours and anthropometry |  |  |  |  |  |  |  |  |
|  |  |  |  |  |  |  |  |  |
| BMI category (2HC) |  |  |  |  |  |  |  |  |
| Underweight | 0 ( 0.0) | 0 ( .) | 0.60 (0.34-1.05) | 0.60 (0.32-1.12) | 13 ( 0.4) | 3 (23.1) | 1.27 (0.35-4.65) | 1.06 (0.22-5.07) |
| Normal Weight | 553 (28.5) | 88 (15.5) | 1 | 1 | 1219 (40.7) | 233 (19.1) | 1 | 1 |
| Overweight | 1103 (56.9) | 121 (16.2) | 1.05 (0.78-1.42) | 1.03 (0.76-1.40) | 1214 (40.5) | 207 (17.1) | 0.87 (0.71-1.07) | 0.82 (0.65-1.03) |
| Obese | 281 (14.5) | 47 (16.5) | 1.08 (0.73-1.59) | 1.07 (0.72-1.58) | 545 (18.2) | 106 (19.4) | 1.02 (0.79-1.32) | 1.04 (0.78-1.37) |
| Missing | 6 (0.1) |  |  |  | 6 (0.1) |  |  |  |
|  |  |  |  |  |  |  |  |  |
| Smoking status (Follow3) |  |  |  |  |  |  |  |  |
| current smoker | 157 ( 8.1) | 19 (12.1) | 0.66 (0.39-1.11) | 0.54 (0.30-0.99) | 214 ( 7.1) | 31 (14.5) | 0.72 (0.49-1.08) | 0.64 (0.41-1.00) |
| former smoker | 1156 (59.6) | 193 (16.7) | 0.96 (0.74-1.24) | 1.00 (0.75-1.34) | 1137 (38.0) | 206 (18.1) | 0.95 (0.78-1.15) | 0.89 (0.72-1.10) |
| never smoked | 618 (31.9) | 107 (17.3) | 1 | 1 | 1626 (54.3) | 308 (18.9) | 1 | 1 |
| Missing | 26 (0.5) |  |  |  | 26 (0.5) |  |  |  |
|  |  |  |  |  |  |  |  |  |
| HSV-2 infection |  |  |  |  |  |  |  |  |
| 0 | 1862 (96.0) | 307 (16.5) | 1 | 1 | 2809 (93.8) | 528 (18.8) | 1 | 1 |
| 1 | 78 ( 4.0) | 14 (17.9) | 1.11 (0.61-2.00) | 1.15 (0.61-2.14) | 185 ( 6.2) | 21 (11.4) | 0.55 (0.35-0.88) | 0.53 (0.32-0.89) |
| *Adjusted for gender, age, ethnicity, SES, lifestyle factors (smoking and BMI) and psychological stress**Additionally adjusted for UV light exposure, fatigue and HSV-2 status | | | | | | | |  |
